# Urinary and cellular volatile organic compounds as biomarkers for urological cancers: a systematic review of GC-MS-based volatolomics

**DOI:** 10.1007/s11306-026-02493-7

**Published:** 2026-07-27

**Authors:** Boyu Xie, Qing Wen

**Affiliations:** 1 Faculty of Biophysics, Biochemistry and Molecular Biology, School of Life Sciences, Tsinghua University, Beijing, 100084 China; 2 Department of Urology, The First Affiliated Hospital, School of Medicine, Zhejiang University, Hangzhou, 310000 China; 3 Department of Surgery and Cancer, Imperial College London, London, W12 0HS UK

**Keywords:** Volatile organic compounds (VOCs), Urological cancers, Gas chromatography-mass spectrometry (GC–MS), Urinary biomarkers, Cellular volatolomics, Cancer diagnosis

## Abstract

**Introduction:**

Volatile organic compounds (VOCs) in human biofluids are promising non-invasive cancer biomarkers. However, their potential in urological cancers, including prostate, bladder, and kidney cancers is less explored, and the relationships between urinary and cellular VOC profiles across these cancer types remain unclear.

**Objectives:**

This systematic review aimed to evaluate gas chromatography-mass spectrometry (GC-MS)-based untargeted volatolomics studies in urological cancers to investigate cancer-specific, cross-cancer, and cross-matrix (urine vs. cell culture media) VOC biomarkers, while assessing methodological factors influencing biomarker discovery.

**Methods:**

Published urinary and cellular VOC studies in prostate, bladder, and kidney cancers were systematically reviewed. VOC biomarkers, direction of change, diagnostic performance, sampling methods, analytical platforms, compound-identification strategies, and statistical approaches were extracted and compared across cancer types and sample matrices.

**Results:**

A total of 12 urinary prostate, 9 urinary bladder, 5 urinary renal, and 3 cellular VOC studies were identified. Prostate cancer profiles were mainly characterized by decreased organic acids and alcohols, whereas bladder and renal cancers more frequently showed elevated ketones and aldehydes. Several VOCs, including 2-butanone, 2-heptanone, hexanal and phenol, were identified across multiple cancer types. Cellular studies showed consistent directional changes for six VOCs, and cross-matrix analysis identified 28 shared VOCs, seven of which showed aligned trends in urine and cell models. These findings suggest that VOCs reflect both unique and shared metabolic alterations in urological cancers and may serve as useful diagnostic biomarker candidates.

**Conclusion:**

This review integrates urinary and cellular VOC data across major urological cancers and identifies unique, recurrent, and cross-matrix VOCs that warrant further mechanistic and clinical validation. Although methodological heterogeneity remains a challenge, VOC-based approaches have generally shown good sensitivity and specificity for distinguishing malignant from healthy controls. With improved and standardized analytical methods, VOC volatolomics may support the future development of non-invasive and accurate diagnostics for urological cancers.

**Supplementary Information:**

The online version contains supplementary material available at https://doi.org/10.1007/s11306-026-02493-7.

## Introduction

### Biological basis and history of volatile organic compound biomarker discovery

Volatile organic compounds (VOCs) are defined as compounds with high vapor pressure and a boiling point < 250 °C at 101.3 kPa (WHO, 1989). VOCs can be produced through various endogenous metabolic processes in human, such as lipid peroxidation, enzyme catabolism, and gut microbiota metabolism (Janfaza et al., 2019; Ruzsanyi et al., 2022). Their profiles can also be altered by pathological conditions such as inflammation, neoplasia, and tumorigenesis, and the disease-associated changes in VOC profiles are detectable in human biofluids, enabling their use as non-invasive biomarkers for monitoring and diagnosis of conditions ranging from infections to malignancies (Janfaza et al., 2019).

The history of profiling VOCs for healthcare applications was initiated by Pauling et al. (1971) who identified 250 VOCs in breath and 280 in urine, which demonstrated unique VOC patterns for tracking metabolic change in human. The first use of gas-chromatography enabled their analysis of “urine headspace” – the gaseous phase above the urine sample (referred to as “vapor” in their study). However, VOCs were not investigated for diagnostic discrimination until 1985, when Gordon et al. differentiated lung cancer patients from healthy controls based on breath volatiles, despite no specific compound being identified due to technological limitations at the time (Gordon et al., 1985).

Modern analytical techniques enable the detection of disease-specific VOCs across various biological matrices, including breath, skin secretions (e.g., sweat, head and neck secretions), saliva, milk, gastric contents, blood (whole blood, serum, and plasma), urine, and feces (de Lacy Costello et al., 2014). VOC biomarkers have therefore been discovered to associate with numerous pathological conditions, such as liver cirrhosis (Fernández del Río et al., 2015), ulcerative colitis, Crohn’s disease, Parkinson’s disease, pulmonary hypertension, pre-eclampsia toxemia, and chronic kidney diseases (Nakhleh et al., 2017). VOCs have also been extensively investigated in cancers. Using breath tests, discriminatory biomarkers have been identified for thyroid (Guo et al., 2015), esophagogastric (Markar et al., 2018), lung (Saalberg & Wolff, 2016), breast (Li et al., 2014), liver (Qin et al., 2010), ovarian (Amal et al., 2015), and colorectal (Altomare, 2013) cancers. Urine tests have also been conducted for head and neck squamous cell carcinoma (Opitz & Herbarth, 2018), lymphoma (Hua et al., 2018), lung (Hanai et al., 2012), cholangiocarcinoma (Navaneethan et al., 2015), colorectal (Arasaradnam et al., 2014) and urological cancers.

### Diagnostic challenges in urological cancers

Urological cancers—primarily prostate, bladder, and kidney (renal) cancers—have high incidence and are associated with significant mortality. Together they account for over 2.4 million annual diagnoses and approximately 750,000 fatalities globally (Ferlay et al., 2021). Additionally, current diagnostic pathways – such as serum prostate specific antigen (PSA) testing, digital rectal exam (DRE), transrectal ultrasonography (TRUS), magnetic resonance imaging (MRI) and biopsy for prostate cancer (Cornford et al., 2024; Litwin & Tan, 2017; Wei et al., 2014), CT urography, intravenous urography, cystoscopy and biopsy for bladder cancer (Gontero et al., 2024), and renal cancer assessment with estimated glomerular filtration rate, ultrasound, CT/MRI and biopsy (Bex et al., 2025; Capitanio & Montorsi, 2016; Kamat et al., 2016) – remains invasive, costly, and susceptible to overdiagnosis with variable sensitivity and specificity (Bex et al., 2025; Cornford et al., 2024; Gontero et al., 2024). Therefore, early diagnosis supported by non-invasive, accurate and standardized biomarker is crucial.

Yet urological VOC research remains relatively under-represented: most VOC biomarker studies have focused on lung, breast, and colorectal cancers, whereas fewer than 1% of usable biomarkers have been reported for urological malignancies (Janfaza et al., 2019). Although the number of identified biomarkers only partly reflects study frequency and does not directly determine clinical utility, the limited number of studies and reported biomarkers indicate a critical need for further biomarker discovery in these cancers.

### Rationale for urinary and cellular VOC biomarker analysis

Urine, a biologically relevant matrix for urological malignancies, is produced, stored and flowed through in the urinary tract (kidneys, bladder) and is in close anatomical proximity to the prostate. Compared with breath, urine generally provides a higher and more stable concentration of volatile analytes, supports long-term storage with fewer pre-analytical losses, easier for sampling and is less susceptible to ambient air contamination (Becker, 2020; Goertzen et al., 2024). These attributes, together with established workflows for standardized collection and handling, suggest that urinary VOCs may yield more reliable biomarker candidates for clinical translation (Becker, 2020; Goertzen et al., 2024). Since urine tests have been applied in fewer studies compared to breath tests, accounting for less than 8% of total VOC biomarkers discovered (Janfaza et al., 2019), this review mainly focuses on urinary VOC biomarker discovery in urological cancers.

To complement biofluid findings, cell-based studies for urological cancers which enable the experiments under controlled and reproducible conditions were also reviewed. Culture parameters – including medium composition, passage number, oxygen tension, and nutrient availability can be standardized, allowing quantification and normalization of VOC emissions that are directly linked to tumor cell metabolism. Such models support targeted perturbations (e.g., oxidative stress, hypoxia) that help delineate pathways underlying candidate biomarkers and strengthen causal interpretation of urine-based findings.

Together, urinary and cellular VOC analyses may offer complementary perspectives: biofluid samples provide non-invasive, patient-friendly biomarker opportunities, while cell culture studies may reveal the underlying metabolic pathways to enable further mechanistic interpretation. Combining both approaches may help identify robust, clinically relevant VOC biomarkers for the early detection and monitoring of urological cancers.

### Analytical approaches and scope of this review

The idea of using VOCs as biomarkers for cancer detection was proved by experiments using trained canine olfaction. Dogs were able to differentiate urine samples from patients with prostate or bladder cancer from those of healthy controls, achieving diagnostic accuracies exceeding 90% (Cornu et al., 2011; Willis et al., 2011). These findings provided an important proof-of-principle that cancer-related odor signatures are present in urine and can be detected biologically. However, canine olfaction provides only a behavioral yes/no classification and does not identify or quantify the specific VOCs responsible for discrimination. It also faces challenges in standardization, training cost and inter-animal variability. Modern alternatives, such as electronic noses (or e-sensors) rely on pattern recognition of raw sensor data, chromatographic fingerprints, sometimes combined with subsequent data analysis for component separation, but they show relatively limited sensitivity and specificity (usually ≤ 75%) and do not identify specific biomarker compounds (Behera et al., 2019; Roine et al., 2012). To not only distinguish cancer from normal samples but also identify the specific VOCs responsible for this separation, mass spectrometry-based techniques are preferred. These methods offer quantitative precision, high sensitivity, and standardized analytical protocols, which all enables robust biomarker discovery and advancing translational research in cancer-associated volatolomics (Janfaza et al., 2019).

Gas chromatography-mass spectrometry (GC-MS) is the predominantly employed technique for VOC analysis and widely regarded as the “gold standard”. While alternative systems such as proton-transfer-reaction MS (PTR-MS) and selected-ion flow-tube MS (SIFT-MS) may be used depending on the sample matrix, phase, and target analytes, GC-MS remains the most commonly adopted approach for untargeted volatolomics studies. A typical GC-MS system comprises four functional modules: (1) a sampling module, which uses adsorbent materials to trap VOCs (although direct injection can bypass this step); (2) a thermal desorption unit, which releases analytes into the gas chromatograph; (3) the gas chromatograph itself, which separates compounds via a capillary column based on properties such as polarity or molecular weight; and (4) the mass spectrometer, which identifies the separated compounds through mass-to-charge (m/z) ratios.

For VOC sampling, most studies employ commercially available techniques such as direct injection, solid-phase microextraction (SPME), thermal desorption (TD), stir-bar sorptive extraction (SBSE), and high-capacity sorptive extraction (HiSorb™). The choice of sampling strategy and analytical platform depends on balancing sensitivity, throughput, and matrix complexity according to specific research aims. These methodological considerations are also summarized in this review.

The purpose of this systematic review is to summarize the published literature on urinary and cellular VOC biomarkers associated with urological cancers, including prostate, bladder, and kidney cancers. To ensure consistency across studies, this review focuses on VOCs analyzed using GC–MS-based or related gas-phase analytical platforms, regardless of whether sampling was performed by headspace or immersive extraction. Specifically, the objectives of this review are: (1) to identify urinary and cellular VOC markers reported for three cancers; (2) to compare VOC profiles across cancer types to distinguish shared and cancer-specific signatures; and (3) to evaluate methodological factors influencing VOC detection, such as sampling techniques and analytical conditions that may affect VOC emission and detection.

## Methods for systematic review of urinary and cellular VOC biomarkers in urological cancers

### Literature search strategy and information sources

The search strategy for this systematic review followed by the Preferred Reporting Items for Systematic Reviews and Meta-Analyses (PRISMA) guidelines. Literature searches were conducted in four main databases: PubMed, Embase, Web of Science and Scopus. PubMed searches included records indexed in MEDLINE and PubMed Central (PMC). These databases were selected to provide broad coverage across biomedical, healthcare, metabolomics, biomarker and analytical chemistry literature. Google Scholar was used as a supplementary source to identify potentially relevant studies not captured by the main database searches, as its ranking algorithm is not fully transparent, search results can vary over time, and the total number of retrievable records is difficult to define reproducibly. Overall, four main databases (PubMed, Embase, Web of Science, and Scopus) and one supplementary source (Google Scholar) were used in this review.

Search terms were constructed using combinations of controlled vocabulary, including Medical Subject Headings (MeSH), and free-text synonyms. Boolean operators were used to combine terms, with “OR” applied within each concept block and “AND” applied between concept blocks. Where appropriate, phrase searching using quotation marks, field tags such as [Title/Abstract], and truncation using “*” were applied. The full PubMed search strategies are provided in Supplementary Table 1. Equivalent search concepts were applied in Embase, Web of Science, and Scopus, with minor database-specific adaptations according to each platform’s search interface, controlled vocabulary, and indexing structure. For databases with built-in search fields such as Scopus, individual keyword blocks were entered separately and combined by choosing platform’s Boolean operators (AND/OR).

Using prostate cancer as an example, the search combined cancer-related terms, such as “prostate cancer”, “prostatic neoplasm”, “prostatic carcinoma”, and “prostate adenocarcinoma”, with VOC-related terms, such as “volatile organic compound”, “volatile organic compounds”, “VOC”, “VOCs”, “volatilome”, “volatolome”, “volatilomics”, “volatolomics”, “volatile metabolite”, and “metabolite”, and sample-related terms, such as “urine”, “urinary”, and “headspace”. For kidney cancer, both “kidney” and “renal” were used to account for differences in terminology. The full search terms for each cancer type and sample matrix are listed in Supplementary Table 1. The search covered publications up to 1 st May 2026, with no date restrictions.

### Eligibility criteria and study screening

All retrieved article titles and abstracts were imported into Zotero software (version 9.0, Zotero.org) using the Zotero browser connector. When automatic import was unsuccessful, records were added manually using the article title or DOI. Duplicate records were identified using Zotero’s duplicate-detection function and then manually checked and removed.

For urinary VOC studies, several inclusion criteria were applied during title and abstract screening: (1) Published work must be original, peer-reviewed research articles written in English. Duplicate records retrieved from different databases were removed, and review articles, editorials and comments were also excluded. (2) Studies were required to include a cancer-normal comparison, excluding those that compared only different cancer types without a healthy control group involved, or that focused on ambient VOC exposure and carcinogenesis. (3) Studies were required to use urine as the sample matrix and report data specific to urological cancers, excluding studies of other cancer types or non-cancer diseases). (4) Only instrument-based, quantitative analytical methods with VOCs identified were included, which rules out those relying on trained canine olfaction or electronic nose, as these do not allow further compound-level identification or quantification. (5) Studies had to use an untargeted analytical approach, as targeted studies generally focus on a small number of pre-selected VOCs from previous findings and often employ different analytical platforms.

For cellular VOC studies, a similar screening strategy was applied. Eligible studies were required to analyze VOCs from urological cancer cell lines or relevant normal control cell lines using instrument-based analytical methods. Non-original research articles, non-cellular studies, studies unrelated to urological cancers, and studies without compound-level VOC identification were excluded.

**Fig. 1:**
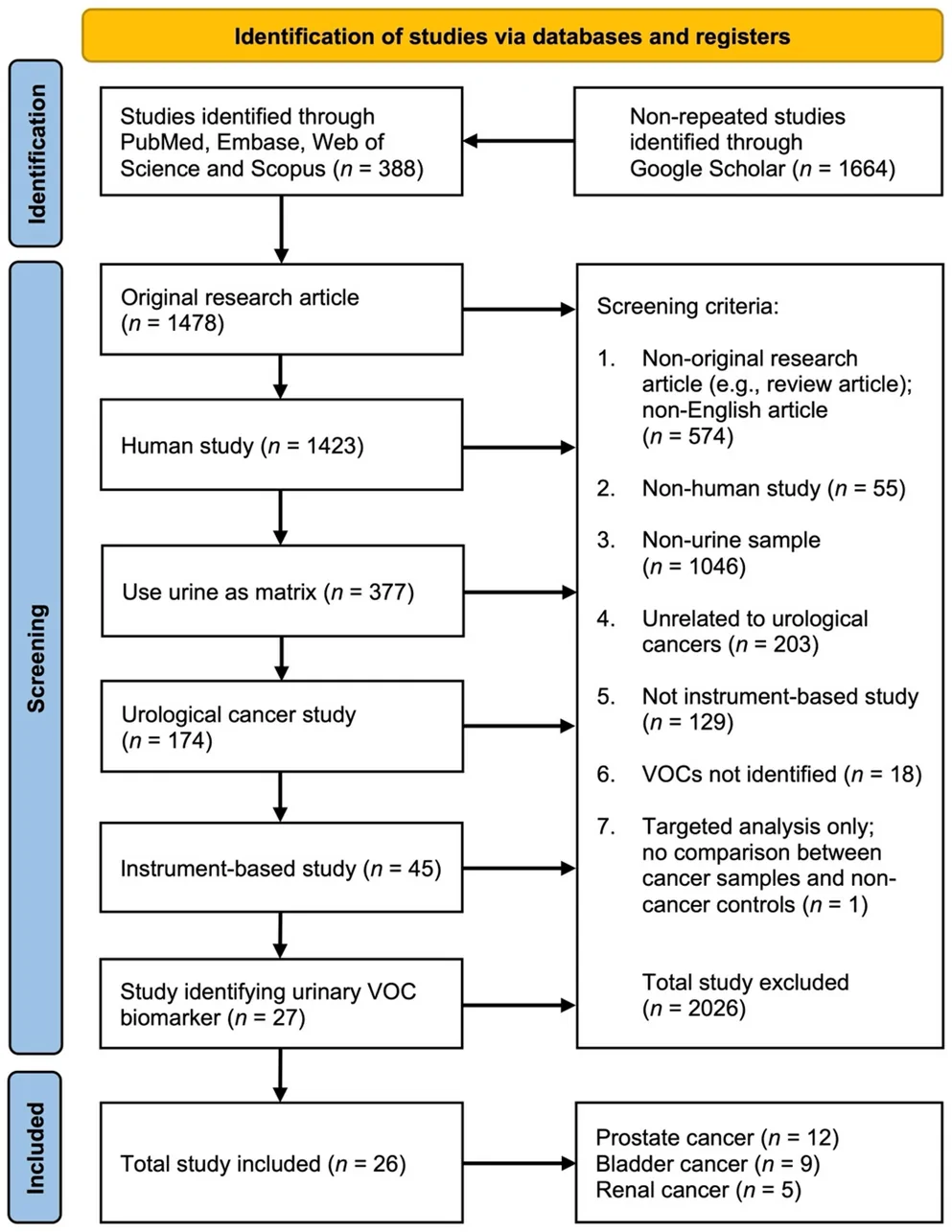
Preferred reporting items for systematic reviews and meta-analysis (PRISMA) flowchart for the systematic review of urinary VOC studies in prostate, bladder, and renal cancers. The diagram illustrates the systematic search process, study identification, and screening criteria applied for the inclusion of urological cancer studies

Screening was conducted independently by two reviewers in a double-blind manner. Discrepancies were resolved through discussion. The final included urinary studies were summarized in the main PRISMA flowchart (Fig. 1). The completed PRISMA checklist is provided in Supplementary Table 2. Detailed cancer-specific urinary PRISMA flowcharts are provided in Supplementary Fig. 1 (A-C). The cellular VOC screening process is summarized separately in Supplementary Fig. 2.

### Methodological quality assessment

Urinary VOC studies involve important procedures of clinical sample collection, patient selection and diagnostic evaluation. Therefore, these studies were further subjected to a methodological quality assessment (risk of bias and applicability) by using the Quality Assessment of Diagnostic Accuracy Studies (QUADAS-2) tool (Whiting et al., 2011). This tool assesses four domains: patient selection, index test, reference standard, and flow and timing. The signaling questions were tailored to VOC diagnostic studies in advance, and the assessment form was piloted before use. Two reviewers also independently evaluated each study, with disagreements resolved by consensus. Domain-level judgements were represented as “low”, “high” and “not reported”, respectively, following QUADAS-2 guidance. The overall results are summarized in Supplementary Table 3.

### Data extraction, VOC biomarker selection and diagnostic model performance

For each eligible study, we first extracted general study and methodological information, including cancer type, study design, sample matrix, cohort size or cell lines used, sampling/extraction method, sorbent or probe coating material, analytical platform, identified VOC biomarkers, direction of change where reported, and diagnostic model performance metrics including accuracy, sensitivity and specificity where available. Methodological variables, including internal standard use, compound identification strategy, QC/blank use, data preprocessing, and statistical analysis, were also recorded (Supplementary Table 4). Information not provided in the original article was marked as “not reported”, and compounds identified without a reported direction of change were marked as “unknown”. Funding sources were not extracted, as they were not directly relevant to the biomarker-focused synthesis.

Each study that met the inclusion criteria was then assessed for the reliability of its reported VOCs. This assessment considered whether the authors applied appropriate quality-control (QC), compound-identification, data-processing and statistical procedures, including: (1) **use of internal standards**, defined as the addition of known reference compounds during sampling or analysis to support data standardization, alignment and comparability across runs; (2) **compound identification methods**, including retention time (RT), retention index (RI), or equivalent approaches; (3) **library matching**, defined as verification of compound identities by comparison with the National Institute of Standards and Technology (NIST) library or other comparable spectral databases; (4) **use of blanks or QC samples** to support baseline normalization, removal of background signals or contaminants, and exclusion of sporadically detected VOCs; (5) **data-preprocessing and feature-screening strategies**, including dataset trimming before analysis, baseline correction, chromatographic alignment, and exclusion of low-confidence or unqualified compounds; and (6) **statistical approaches**, including multivariate and univariate analyses, with specific statistical tests recorded where reported (Supplementary Table 4).

We also extracted whether the original studies reported adjustment for multiple VOC comparisons, including false discovery rate (FDR), Bonferroni correction, or related procedures. Feature-filtering procedures, *p*-value thresholding, LASSO/logistic regression, machine-learning algorithms and model-validation strategies were recorded where available, but these were considered separately from formal multiple-comparison correction. These details are also summarized in Supplementary Table 4.

For studies that passed reliability assessment, detailed VOC biomarker data were extracted and analyzed in this review. Only VOCs showing statistically significant differences between cancer and normal samples were included. Compounds used solely for differentiating between cancer types were excluded. One study from Lima et al. (2020) primarily focused on the differential diagnosis of prostate cancer from other urological cancers rather than a direct cancer–normal comparison. However, as cancer–normal comparison data were available in the supplementary materials, VOCs showing significant differences between cancer and control samples were included in our analysis.

Because the included studies differed substantially in sample preparation, analytical platforms, data preprocessing, and statistical modelling, no quantitative meta-analysis or pooled effect measure was calculated. Results were synthesized descriptively using the reported direction of VOC change, recurrence frequency across studies, cross-cancer and cross-matrix overlap, and diagnostic performance metrics reported by the original studies.

Where studies reported diagnostic model performance, sensitivity, specificity, and accuracy were extracted and reported as percentages without decimal places; for example, 93.5% was rounded to 94%. Although most of the studies employed threshold-independent metrics such as area under the curve (AUC) in ROC analysis, it should be noted that these reported accuracies are not suitable for cross-study comparisons. Where validation cohort results were available, these were preferentially reported over training cohort results. For studies reporting multiple diagnostic models, the best-performing model was recorded and indicated as “≤” the reported value where appropriate. Where studies reported a range of model performances, this range was retained (e.g., A%-B%).

### Chemical name harmonization

Compound names were standardized to improve consistency across studies. Because VOC studies often report compounds directly from NIST library outputs, compound names in this review were harmonized primarily according to commonly used NIST and PubChem names. Where possible, the International Union of Pure and Applied Chemistry (IUPAC)-style names were also noted where appropriate to improve chemical clarity. For studies that reported compounds only using IUPAC nomenclature, names were manually converted to the corresponding NIST/PubChem-style names and then checked against NIST and PubChem records.

For example, the NIST-style inverted name “2-Hexanol, 2-methyl-” was converted to the non-inverted form “2-Methyl-2-hexanol” for consistency and readability. The strict IUPAC-style name for this compound is “2-methylhexan-2-ol”; however, many VOC and metabolomics studies use NIST- or PubChem-style names rather than strict IUPAC nomenclature. Therefore, commonly adopted NIST/PubChem compound names were retained where appropriate, so that readers can more readily compare newly reported VOC biomarkers with those summarized in this review. Synonyms were checked against NIST and PubChem records where necessary. Where spelling variants occurred, such as “sulphide” and “sulfide”, the American spelling “sulfide” was used consistently.

## Results

### Urinary VOC biomarker profiles in prostate, bladder and renal cancers

We first summarized the findings for VOC biomarkers identified in prostate, bladder and renal cancers individually. In total, 12 prostate cancer studies, nine bladder cancer studies, and five renal cancer studies were included and are summarized in Table 1. All listed compounds were reported as significantly altered in the urine of cancer patients compared to healthy controls. Compounds identified but not showing significant differences were excluded. Where authors have quantified the peak values of certain compounds, these have been denoted as “increase” or “decrease” to indicate whether compound levels were higher or lower in cancer samples relative to controls. For studies that identified compounds but did not compare levels, these compounds are marked as “unknown” in the tables to reflect the lack of comparative data. Where reported, the accuracy, sensitivity and specificity were also summarized in Table 1.

**Table 1:** Summary of 12 prostate cancer, nine bladder cancer and five renal cancer VOC biomarker studies

Study	No. of patient (controls: cancer)	Sampling method	Instrument platform	Identified biomarkers	Accuracy (%), (sensitivity%: specificity%)
**Prostate Cancer Studies**					
Smith et al. (2010)	24:13	Headspace CAR/PDMS SPME	GC-MS	**Unknown**: 2-Octanone, Acetaldehyde, Butyrolactone, Methyl propyl disulfide, Methyl vinyl ketone	(92%:96%)
Khalid et al. (2015)	43:59	Headspace CAR/PDMS SPME	GC-MS	**Unknown**: 2,6-Dimethyl-7-octen-2-ol, 2-Octanone, 3-Octanone, Pentanal	≤ 74%
Struck-Lewicka et al. (2015)	32:32	Headspace direct sampling	LC-ESI-TOF-MS GC-MS	**Decreased**: 2-Keto-l-gluconic acid, Acetic acid, Aconitic acid, Arabitol, Butyric acid, Galactaric acid, Hippuric acid, HPHPA (Hydroxyphenylhydroxypropionic acid), Indole, Inositol, Isobutyric acid, Isocitric acid, meso-Erythritol, Propanetricarboxylic acid, Propenoic acid, Propionic acid, Threonic acid	Not reported
Jiménez-Pacheco et al. (2018)	21BPH:29	Headspace SPME	GC-MS	**Decreased**: 2-Butanone, 3-Methylphenol, Furan, Phenol, p-Xylene	Not reported
Gao et al. (2019)	Training 53:55 Test 22:53	Immersive PDMS SBSE	GC-MS	**Unknown**: 1-(2,4-Dimethylphenyl)-3-(tetrahydrofuryl-2)propane, 1,1,1,5,5,5-Hexamethyl-3,3-bis[(trimethylsilyl)oxy]-trisiloxane, 1,1,3,3,5,5,7,7,9,9-Decamethyl-pentasiloxane, 1-Propylpentachlorotriphosphazene, 2,6-Ditbutyl-4-hydroxymethylene-2,3,5,6-detetrahydrocyclohexanone, 2-Aminoimidazole-5-carboxylic acid, 4-(3,4-dihydro-2,2,4-trimethyl-2 H-1-benzopyran-4-yl)-Phenol, 4-Nitro-4-chlorodiphenylsulfoxide, Estradiol, Ethylhydroxymyristate trisiloxane, Phthalic acid, bis(7-methyloctyl) ester	86%
Lima et al. (2019)	Training 40:42 Test 18:18	Headspace CAR/DVB/PDMS SPME	GC-TQ-MS	**Increased**: 2,5-Dimethylbenzaldehyde **Decreased**: p-Menth1-en-8-ol, 2,2,2,8a-Tetramethyl-3,4,4a,5,6,8a-hexahydro-2 H-chromene, 2,2,7,7-Tetramethyltricyclo[6.2.1.0¹,6]undeca-3,5,9-triene (4,5,9,10-dehydroisolongifolene, 2,6,6,10-Tetramethyl-1-oxaspiro[4.5]dec-9-ene, 2,6-Dimethyl-6-hepten-2-ol, 2-Hexanone, 2-Hydroxy-2-methylphenylpropanone, 2-Methylcyclopentanone, 3,4-Dimethylcyclohex-3-ene-1-carbaldehyde, 3-Carene, 3,7-Dimethylocta-1,6-dien-3-ol, 3-Methyl-6-isopropylidenecyclohexene, 4,6-Dimethyl-2-heptanone, 4-Methyl-1-propan-2-ylcyclohex-3-en-1-ol, 4-Methyl-1-decene, 4-Methyl-3-hexanone, 5-Methyl-2-isopropycyclohexyl acetate L1, 5-Methyl-2-heptanone, Cycloalkenes, Hexanal	86%, (89%:83%)
Lima et al. (2020)	20:20	Headspace CAR/DVB/PDMS SPME	GC-SQ-MS	**Increased**: Hexanal, 2,5-Dimethylbenzaldehyde, 3-Phenylpropionaldehyde, Ethylbenzene, Methyl benzoate **Decreased**: 3-Methyl-benzaldehyde, 4-Methyl-3-hexanone, Dihydroedulan IA, 2-Heptanone, Methylglyoxal	89%, (78%, 100%)
Guest et al. (2021)	38:12	Headspace CAR SPME	GC-MS	**Increased**: 2,5-bis[(trimethylsilyl)oxy]-Benzaldehyde, 3,3,5,5,7,7,9,9,11,11,13,13-Dodecamethyl-1,15-bis(1,3,3,5,5-pentamethyl-2,4,6-trioxa-1,3,5-trisilacyclohexyl-4,6,8,10,12-tetraoxa-3,5,7,9,11-tetrasilapentadecane, Trimethyl-Silanol **Decreased**: 1-(ethenyloxy)-Octadecane, 2-Pentanone	94%
Tyagi et al. (2021)	36:55	Headspace TD	GC-IMS / GC-TOF-MS	**Unknown**: Toluene, 2-Ethyl-1-hexanol, 2-Methylcyclopentanone, Acetic acid, Dimethyl disulfide, Phenol, Pyrrole	94%, (78%: 88%)
Liu et al. (2023)	Training 64:43 Test 23:23	Headspace direct sampling	GC-IMS	**Increased**: Furan-3-methanol **Decreased**: (E, E)-Octadeca-2,4-dienal, 2-Ethylhexan-1-ol, 2-Undecen-1-al	96%
Riccio et al. (2023)	30:26	Headspace CAR/DVB/PDMS SPME	GC-MS	**Unknown**: 3,5-Dimethylbenzaldehyde, 1,1,6-Trimethyl-1,2-dihydronaphthalene, 2,5,5,8a-tetramethyl-1,2,3,5,6,7,8,8-octahydro-1-naphthalenyl ester acetate, 2-Bromophenol, D-Carvone, α-Methylcinnamaldehyde	87%-97%
Badmos et al. (2024)	247:139	SBSE	GC-MS	**Unknown**: 1-Decene, 1,1,3,3,5,5,7,7-Octamethyl-7-(2-methylpropoxy)tetrasiloxan-1-ol, 1,2-Propanediamine, 2-phenyl-3-decyn-1-ol, 2,2-bis(hydroxymethyl)propane-1,3-diol, 2,3-dimethylbutane, 2,5-Di-tert-butyl-1,4-benzoquinone, 3,5-di-tert-Butyl-4-hydroxybenzyl alcohol, 5-ethyl-1,2,3,4-tetrahydronaphthalene, 7 H-Benzo[c]carbazole, Acetophenone, Butane-1,4-diol, Clionasterol, Decamethylcyclopentasiloxane, Decanal, Dodecamethylcyclohexasiloxane, Ethane-1,2-diol, Galaxolide, L-Phenylephrine, Lactose, Methyl dihydrojasmonate, Methyl Glyphosate, N,N-Diisopropylethylamine, Norcodeine, N-trimethylsilyl-, trimethylsilyl ether, Octanal, 2-(phenylmethylene)-, Pipobroman, Propyl 4-nitrobenzoate, Semicarbazide, Tridecane, Versalide	88%-97%
**Bladder Cancer Studies**					
Jobu et al. (2012)	7:9	Extraction needle	GC-MS	Increased: (S)-2-Hydroxypropanoic acid, (Z)-2-Decenal, (Z)-2-Nonenal, 1,2-Benzenedicarboxylic acid, Butyl decyl ester, 4,5-Dimethyl-3(2 H)-isoxazolone, Diethyl phthalate, Dodecanal, Ethylbenzene, Nonanoyl chloride, Pentadecanoic acid, Pentanoic acid 4-methyl-1-buten-1-yl ester, Trichloroacetic acid 3-tridecyl ester	Not reported
Cauchi et al. (2016)	187:72	Headspace CAR/PDMS SPME	GC-TOF-MS	**Increased**: 2-Butanone, 3-Hydroxyanthranilic acid, Benzaldehyde, Benzoic acid, Butyrophenone, cis-3-Hexanoic acid, Hexanal, trans-3-Hexanoic acid **Decreased**: 2,3-Butanedione, 2-Pentanone, 2-Propanol, 4-Heptanone, Acetic acid, Dimethyl disulfide, Piperitone, Thujone	89% (90%: 88%)
Pinto et al. (2021a, b)	56:53	Headspace CAR/DVB/PDMS SPME	GC-MS	**Increased**: 1,2,4-Trimethylbenzene, 1-Methylnaphthalene, 2,4-Dimethylheptane, 2,6-Dimethylnonane, 2-Methylnaphthalene, 2-Methylnonane, 4-Methyloctane, p-Cresol **Decreased**: (1 S,5R)-1,5-dimethyl-6,8-dioxabicyclo[3.2.1]octane, 2-Butanone, 2-Furaldehyde, 2-Methylbutanal, 4-Heptanone, Carvone, Formaldehyde, Hexanal, Piperitone	80% (70%:89%)
Tyagi et al. (2021)	36:15	Headspace TD	GC-IMS / GC-TOF-MS	**Unknown**: 2-Pentanone, 4-Heptanone, Benzoic acid, Biphenyl, Dodecane, Heptanal, Hexadecane, Methyl Isobutyl Ketone, Methylglyoxal, Naphthalene, Nonanal, 2,6,10,14-Tetramethylpentadecane, Undecane	82% (27%:94%)
Lett et al. (2022)	220:110	Headspace CAR/DVB/PDMS SPME	GC-MS	**Increased**: 5-Ethyl-3-methyl-2(5 H)-furanone, 2-Methoxyphenol, 3-Methyl-2-heptanone, 4-Methyl-3-pentenoic acid, 2-Heptanone, Phenol **Decreased**: 1,1,4a-Trimethyl-4,5,6,7-tetrahydro-3 H-naphthalen-2-one, 1,2,4,5-Tetramethylbenzene, 2-Ethyl-1-hexanol, Nonanal	72% (71%:72%)
Ligor et al. (2022)	57:40	Headspace CAR/PDMS SPME	GC×GC-TOF-MS	**Increased**: 1-(2,6,6-trimethyl-1-cyclohexenyl)-2-Buten-1-one, 1-(2,6,6-trimethylcyclohexa-1,3-dien-1-yl)-2-Buten-1-one, 2,4-Dimethylbenzaldehyde, 2,5-Dimethylbenzaldehyde, 2,6,6-Trimethylcyclohexa-1,3-diene-1-carbaldehyde, 2-Ethyl-3-methoxypyrazine, 2-Methoxyphenol, 2-Pentanone, 3-(methylthio)Propionaldehyde, 3-Methoxy-5-methylphenol, 3-Octen-2-one, 4-(2,6,6-trimethyl-1-cyclohexenyl)-2-Butanone, 4-Ethylphenol, 4-Ketoisophorone, 4-Methyl-2-pentanone, 4-Methylanisole, 6,10-Dimethyl-5,9-undecadien-2-one, Benzyl acetate, Benzyl alcohol, Butyrolactone, Cyclohexanone, Diphenylmethanone, γ-Dodecalactone, Indole, Nerolidol, Nootkatone, Phenylpropylpyridine, Tetrahydro-4-methyl-2-(2-methyl-1-propenyl)pyran **Decreased**: 2-Methoxy-4-vinylphenol, 4-Heptanone, Decanal, Dimethyl disulfide	Not reported
Heers et al. (2024)	30:30	Direct headspace extraction	MCC-IMS	**Unknown**: Benzaldehyde, Benzofuran, Ammonia, Toluene, Hexylbenzene, Cyclohexene 1-methyl-4-(1-methylethylidenem Acetyl valeryl	95% (90%: 100%)
Mao et al. (2025)	67:89	Direct headspace extraction	GC-IMS	**Increased**: 2-Pentanone, Acetone, 2-Butanone, 3-Methylthio-1-propene, Cyclopentanone-D, 2-Heptanone-D, 2-Heptanone, Pentanal-D, Propyl acetate-D, Octanal, (E)-2-Hexen-1-ol, Nonanal, 3-Methylthio-1-propanol, Ethyl salicylate, 2-Undecenal, 1-Hexanol, 2-Acetylpyrazine **Decreased**: 2-Ethyl-1-hexanol, 2-Ethylhexanol-D, Acetic acid	96% (88%:95%)
Carapito et al. (2026)	90:87	Headspace SPME	GC-TQ-MS	**Increased**: 2-Ethyl-1-hexanol, 2,5-Dimethylbenzaldehyde, 4-Methyl-2-heptanone, 4-Methyl-2-pentanone, Hexanal, Nonanal, p-Cresol **Decreased**: Acetone	87%
**Renal Cancer Studies**					
Wang et al. (2016)	25:22	Headspace CAR/PDMS SPME	GC-MS	Increased: Phenol, 1,6-Dioxacyclododecane-7,12-dione, 1-Bromo-1-(3-methyl-1-pentenylidene)-2,2,3,3-tetramethyl-cyclopropane, 2,6-Ditertbutyl-1,4-benzoquinone,2,6-bis(1,1-dimethylethyl)-, 2,6,10,14-Tetramethylpentadecane, 3-Ethyl-3-methylheptane, Aniline, Decanal, Isolongifolene-5-ol, Nonanal, Tetradecane Decreased: Styrene, 4-Heptanone, Dimethylsilanediol	Not reported
Monteiro et al. (2017)	37:30	Headspace DVB/PDMS SPME	GC-IT-MS	**Increased**: Methylglyoxal, Butyl pivalate **Decreased**: 2,5,8-Trimethyl-1,2,3,4-tetrahydro-1-naphthalenol	92 (91%:93%)
Pinto et al. (2021a, b)	75:75	Headspace CAR/PDMS SPME	GC-SQ-MS	**Increased**: 4-Methyl-2-heptanone, Benzaldehyde, Diacetylbenzene, Octanal, *p*-Cresol **Decreased**: 2-Butanone, 2-Furaldehyde, 3-Methylbutanal, 4-Heptanone, Carvone, Hexanal, Methylglyoxal, Naphthalene, Nonanal, p-Mentha-1,5-dien-8-ol, Propanal	81% (80%:82%)
Einoch Amor et al. (2023)	130:101	Headspace in-tube extraction (ITEX)	GC-MS	**Unknown**: 2-Heptanone, Decane, Dodecane, Ethanone, Octane, Pentanone	82% (78%:20%)
Holbrook et al. (2024)	Training 31:163 Testing 12:70	Immersive PDMS SBSE	GC-MS	**Increased**: 2-Ethyl-1-hexanol, Heptadecanolide, cis-Vaccenic acid, 3-Methyldecane, 1,2,3,5,6,7,8,8a-Octahydro-1,8a-dimethyl-7-(1-methylethenyl)-[1R-(1α-7β-8α)]-naphthalene, 1,4-Bis(trimethylsilyl)benzene, Decyl nonyl carbonic acid, L-Ascorbic acid 2,6-dihexadecanoate, (S)-6-Ethenyl-6-methyl-1-(1-methylethyl)-3-(1-methylethylidene)-cyclohexene, 2-Methyl-6-(p-tolyl)hept-2-en-4-ol **Decreased**: 2-Ethylhexylmethylisophthalate, (1α-2β-5α)-5-Methyl-2-(1-methylethyl)-cyclohexanol, 5-Methyl-2-(1-methylethyl)-cyclohexanol, Tributyl ester-1-Propene-1,2,3-tricarboxylic acid, 1,3,5-Triphenyl-cyclohexane, (R)-1-Methyl-4-(1,2,2-trimethylcyclopentyl)-benzene, Isopropylamine, γ-Dodecalactone, Cadala-1(10),3,8-triene, 4-Methylamino-2(5 H)-furanone, 1,2-Dichloro-4-methylbenzene, N-Methylphenethylamine, l-Guanidinosuccinimide	94% (86%:92%)

The studies are listed chronologically from the oldest to the most recent. The table includes details of study design, number of patients, sampling method (including probe-coating materials), analytical platforms, identified biomarkers and model sensitivity, specificity, and accuracy (where reported). In the “Identified biomarkers” column, compounds are grouped according to their reported direction of change in cancer samples relative to controls: “Increased”, “Decreased”, or “Unknown” where the original study did not report the direction of change. Compounds are organized in alphabetical order

CAR (Carboxen); DVB (Divinylbenzene); ESI (electrospray ionization); GC (gas chromatography); IMS (ion mobility spectrometry; LC (liquid chromatography); MS (mass spectrometry); PDMS (Polydimethylsiloxane); SBSE (stir bar sorptive extraction); SPME (solid-phase microextraction); SQ (single quadrupole), TOF (time-of-flight), TQ (triple quadrupole)

**Fig. 2:**
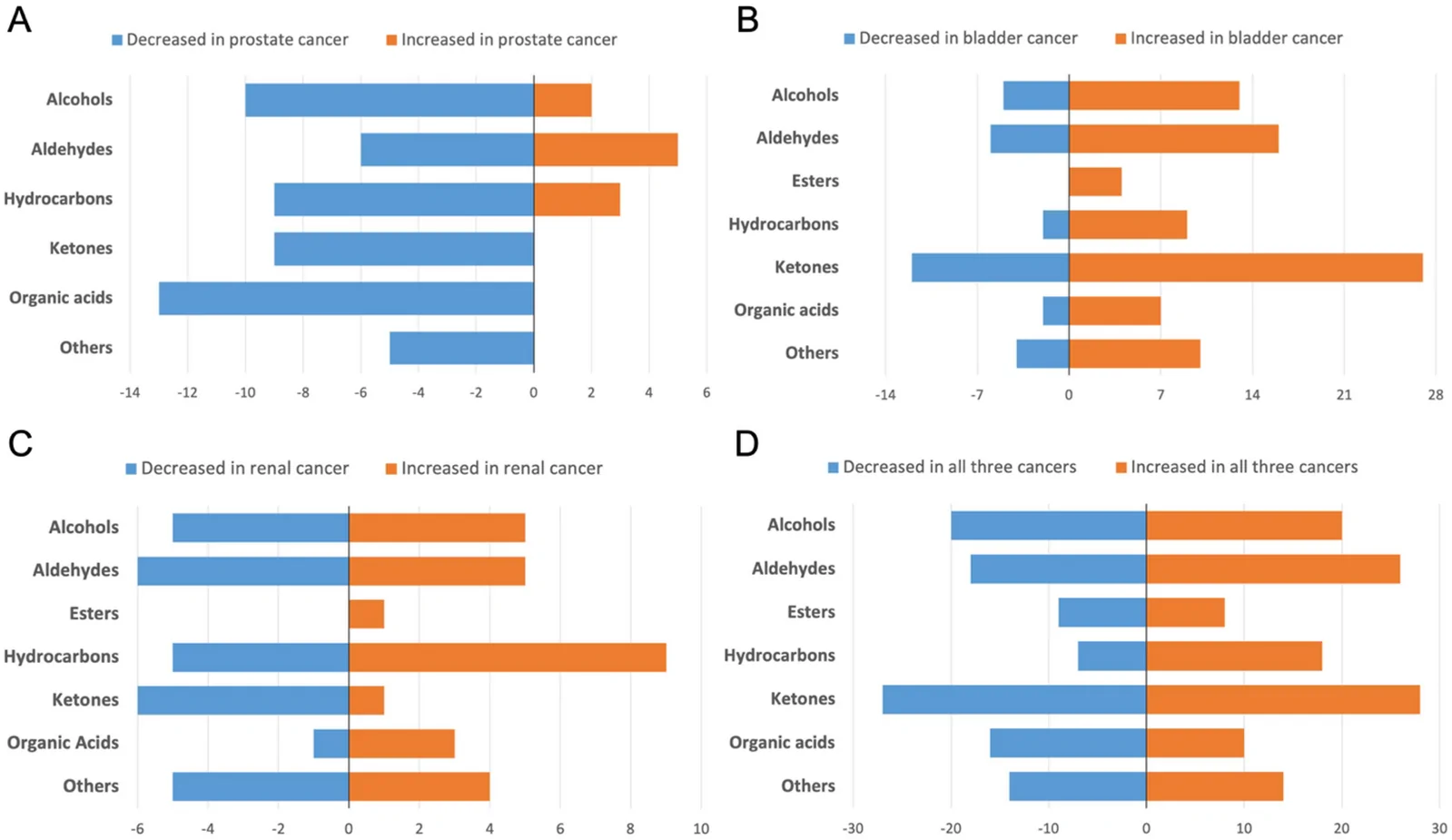
Distribution of volatile organic compound classes identified in urinary studies of **A** prostate, **B** bladder, and **C** renal cancers, and **D** combined analysis of all three urological cancers. Bars represent the number of compounds reported to be increased (orange) or decreased (blue) in cancer compared with healthy controls. Compound classes include alcohols, aldehydes, hydrocarbons, ketones, organic acids, esters, and others

Across all included studies, a total of 117 unique urinary VOC biomarkers were identified for prostate cancer, 108 for bladder cancer, and 59 for renal cancer, relative to their non-cancer controls. For each cancer type, we further analyzed the compound classes among those VOCs reported as either “increased” or “decreased” in cancer samples compared to controls to facilitate the categorization of compound types. Compound classes can be broadly categorized as alcohols (-OH group), aldehydes (-CHO), hydrocarbons (C-H backbone), ketones (C = O), organic acids (-COOH), and others. A few compounds (e.g., dimethyl disulfide, indole, phenol) were less abundant and did not fit into the broader categories, and were therefore classified as “others”. The numbers of each compound class reported as increased or decreased in prostate, bladder and renal studies are summarized in Fig. 2.

In prostate cancer, the majority of identified VOC biomarkers (*n* = 52) were found at decreased levels compared to controls, particularly organic acids, alcohols, hydrocarbons (mainly alkanes and alkenes) and ketones (Fig. 2A). Only ten compounds were reported as significantly increased. This trend aligns with the findings of Wen et al. (2020) (Supplementary Fig. 3). These patterns suggest that VOC alterations may reflect metabolic changes specific to prostate cancer patients.

In contrast, most of biomarkers identified for bladder cancer were increased rather than decreased in cancer samples (86 increased vs. 29 decreased) (Fig. 2B). Ketones were the most frequently altered compound type, with 27 reported as increased and 12 as decreased in cancer. Aldehydes also showed significant changes, followed by alcohols and hydrocarbons. Interestingly, organic acids were less frequently altered and esters showed only mild increased number in bladder cancer, which differs from the pattern seen in prostate cancer.

For renal cancer, an equal number of compounds were identified as increased and decreased (28 of them). Alcohols and aldehydes displayed similar number of compounds between increased and decreased levels, while hydrocarbons were more commonly found at higher levels in cancer samples compared to controls (Fig. 2C). In contrast, ketones were predominantly found in controls.

When combining all three cancers, the overall number of VOCs identified as increased or decreased was roughly equal (Fig. 2D). This indicates that further, more detailed investigation is needed to determine which specific biomarkers have the strongest potential to detect cancer within each cancer type and across urological cancers as a whole.

### Recurrent urinary VOC biomarkers within each cancer type

Within each cancer type, the same VOC biomarkers were reported by multiple studies. To identify the most frequently reported compounds for each cancer type, regardless of whether they were increased, decreased, or reported with an unknown direction of change, we calculated the recurrence frequency of each compound. Recurrence frequency was defined as the number of studies reporting a given compound divided by the total number of studies included for that cancer type (Table 2).

**Table 2:** Summary of recurrent VOC biomarkers identified across all included studies

Compound name	Repeat time/total study number
**Prostate**	
2-Ethyl-1-hexanol	2/12
2-Methylcyclopentan-1-one	2/12
2-Octanone	2/12
2,5-Dimethylbenzaldehyde	2/12
4-Methyl-3-hexanone	2/12
Acetic acid	2/12
Hexanal	2/12
Phenol	2/12
**Bladder**	
2-Pentanone	4/9
4-Heptanone	4/9
Nonanal	4/9
2-Ethyl-1-hexanol	3/9
2-Butanone	3/9
2,5-Dimethylbenzaldehyde	3/9
Hexanal	3/9
2-Heptanone	2/9
2-Methoxyphenol	2/9
4-Methyl-2-pentanone	2/9
Acetic acid	2/9
Acetone	2/9
Benzaldehyde	2/9
Benzoic acid	2/9
Dimethyl disulfide	2/9
Piperitone	2/9
*p*-Cresol (4-methylphenol)	2/9
**Kidney**	
4-Heptanone	2/5
Methylglyoxal	2/5
Nonanal	2/5

For each cancer type, compounds are listed together with the number of studies in which they were reported and their recurrence frequency. Compounds are ranked in descending order of reporting frequency; where compounds have the same frequency, they are arranged alphabetically

For prostate cancer, eight compounds: 2-Ethyl-1-hexanol, 2-Methylcyclopentan-1-one, 2-Octanone, 2,5-Dimethylbenzaldehyde, 4-Methyl-3-hexanone, Acetic acid, Hexanal and Phenol, were all reported in two separate studies, suggesting potential relevance as prostate cancer biomarkers. 17 compounds were repeatedly reported across different bladder cancer studies. Among these, 2-Pentanone, 4-Heptanone and Nonanal were each noted in four individual studies, making them the most frequently reported compounds. 2-Ethyl-1-hexanol, 2-Butanone, 2,5-Dimethylbenzaldehyde and Hexanal appeared in three studies, while the remaining compounds were each documented twice. For renal cancer, 4-Heptanone, Methylglyoxal and Nonanal appeared in two independent studies, indicating possible significance as kidney cancer biomarkers.

Given the limited overlap between studies, these recurrent VOCs should be regarded as preliminary biomarker candidates rather than definitive markers, and they warrant further validation in larger, standardized cohorts. Furthermore, employing multiple analytical platforms and instruments across these studies enhances the reproducibility of their findings, thereby reinforces the reliability of the identified VOCs as candidate biomarkers for each specific cancer type.

### Shared urinary VOC biomarkers across cancer types

Interestingly, compounds that repeatedly appear in one cancer type may also present in others. For example, 4-Heptanone, Acetic acid, Hexanal and Nonanal were all identified in multiple studies across two cancer types (Table 2), suggesting their potential as useful clinical biomarkers with shared relevance across the urinary system.

Although the frequency with which certain compounds are repeated across studies does not necessarily correlate with their true value as biomarkers, it remains informative to examine these recurring compounds. Variations in analytical methods, platforms, sensitivity, and sampling techniques can all affect compound detection, and VOCs that do not appear repeatedly may still hold significant diagnostic value. Nevertheless, identifying “common biomarkers” across urological cancers can provide insight into shared metabolic pathways and help prioritize targets for further validation.

To explore this, all VOCs identified across the 26 included studies were re-analyzed to identify overlapping compounds across cancer types, regardless of whether they were reported as increased, decreased, or unknown. In total, 259 unique VOCs were identified as significantly different between normal and cancer samples. Overlapping compounds found in more than one cancer type were compared and summarized (Table 3).

**Table 3:** Cross-cancer comparison of VOC biomarkers reported in multiple urological cancer studies

Prostate	Bladder	Kidney	Frequency
**Shared compounds across prostate, bladder and renal cancers**			
	Hexanal		2 + 3+1
	2-Ethyl-1-hexanol		2 + 3+1
	2-Butanone		1 + 3+1
	2-Heptanone		1 + 2+1
	Phenol		2 + 1+1
	Carvone		1 + 1+1
	Decanal		1 + 1+1
	Methylglyoxal		1 + 1+2
	Octanal		1 + 1+1
**Shared compounds between prostate and bladder cancers**			
2,5-Dimethylbenzaldehyde			2 + 3
2-Pentanone			1 + 4
Acetic acid			2 + 2
Dimethyl disulfide			1 + 2
Ethylbenzene			1 + 1
2-Undecenal			1 + 1
Butyrolactone			1 + 1
Indole			1 + 1
**Shared compounds between bladder and renal cancers**			
		4-Heptanone	4 + 2
		Nonanal	4 + 2
		*p*-Cresol	2 + 1
		Benzaldehyde	2 + 1
		2,6,10,14-Tetramethyl-pentadecane	1 + 1
		2-Furaldehyde	1 + 1
		4-Methyl-2-heptanone	1 + 1
		Dodecane	1 + 1
		Naphthalene	1 + 1
		*γ*-Dodecalactone	1 + 1

The table lists VOCs identified as biomarkers in more than one study and across three urological cancers. Compounds are grouped according to the cancer types in which they were reported: shared across all three cancers, shared between prostate and bladder cancers, and shared between bladder and renal cancers. Within each group, compounds are ranked in descending order of total reporting frequency; where compounds have the same frequency, they are arranged alphabetically

Intriguingly, nine compounds – 2-Butanone, 2-Ethyl-1-hexanol, 2-Heptanone, Carvone, Decanal, Hexanal, Methylglyoxal, Octanal and Phenol – were universally identified as urinary biomarkers in all three urological cancers across different studies. This may indicate that these compounds are involved in common metabolic processes in urological cancers.

Among the compounds found in two cancer types, eight were shared between prostate and bladder cancers, and ten were shared between bladder and kidney cancers. No compound was identified in both prostate and renal cancer urine samples. This interesting finding may partly reflect anatomical and physiological relationships within the urinary tract. The kidney and bladder are directly connected through urine production, transport and storage, which may contribute to shared urinary VOC alterations. The prostate does not produce or store urine, but it is anatomically adjacent to the bladder and surrounds the proximal urethra; therefore, prostate-associated metabolic or inflammatory changes may still influence urinary VOC profiles.

Overall, this comparison suggests that anatomically or functionally related organs, such as the prostate and bladder or the bladder and kidney, tend to share more recurrent urinary VOC biomarkers. In contrast, the absence of shared VOCs between prostate and kidney cancers may reflect greater differences in tissue function, anatomical distance, and tumor-associated metabolic pathways. However, this interpretation should be treated cautiously because the number of included studies remains limited and methodological heterogeneity may also influence the observed overlap.

Across the 27 overlapping compounds, aldehydes and ketones are the most abundant overlapping biomarkers, followed by hydrocarbons and alcohols, together constituting 87.5% of the total (Supplementary Fig. 4), and is consistent with the previous review (Wen et al., 2020).

### Cellular VOC biomarkers in urological cancer models

The systematic search and screening process identified three cellular VOC studies, one each for prostate, bladder, and kidney cancer cell lines. Zimmermann et al. (2007) initiated the first cellular VOC study on a colorectal cancer cell line by extracting headspace VOCs from cell culture media. Subsequent studies have used headspace extraction method and investigated cellular VOC biomarkers in lung, bronchial (Filipiak et al., 2010), melanoma (Kwak et al., 2013), hepatocellular carcinoma (Mochalski et al., 2013), and gastric (Zhang et al., 2014) cancer cell lines. However, few studies have focused on urological cell lines.

Therefore, we also summarized the VOC extraction methods and conditions and instrumental platforms, which may be beneficial for future mechanistic studies (Table 4). As these studies optimized sampling conditions as part of their protocols, the VOC yield from different conditions were all included in analysis. However, only the biomarkers highlighted by the original authors are presented in the table, while all identified VOCs were included in the subsequent analysis.

**Table 4:** Summary of VOC biomarker studies in prostate, bladder, and kidney cell lines

Study	Cell lines	Cell culture media	Extraction methods & Instrument platform	Extraction condition	Identified biomarkers (regardless testing conditions)
Prostate: Lima et al. (2018)	**Cancer**: PC3, 22RV1, DU145, LNCaP	RPMI-1640	HS-SPME (DVB/CAR/PDMS) GC - SQ - IT - MS	2 ml sample + 0.59 g NaCl, pH = 2 & 7 11 min incubation 30 min extraction (44 °C, 250 rpm)	**Increased**: p-Xylene, 1-Ethoxypentane, 3-Methylbut-3-en-2-ol, 4-Methyl-nonanoic acid, 1-Decanol, Decanoic acid, Methyl nonanoate, Naphthalene, 2-Pentadecanone, Phenylmethanol **Decreased**: 1-(3,5-Dimethyl-furan-2-yl)ethenone, 1,3-Benzothiazole, 2-(4-Methylcyclohex-3-en-1-yl)-2-propanol, 2,7-Dimethyloctan-1-ol, 2-Ethoxy-2-methylbutane, 2-Methylpentane-1,3-diol, 3,7-Dimethyloct-7-en-1-ol, 4-Methyl-benzaldehyde, 4-Methyl-2-heptanone, 4-Methyl-3-penten-2-one, 5-Methyl-2-isopropylcyclohexanol, 5-Methyl-2-heptanone, 6-Pentyltetrahydro-2 H-pyran-2-one, Benzyl acetate, Cyclohexanone, Methyl benzoate, Phenylethanol
	**Normal**: PNT2				
Bladder: Rodrigues et al. (2018)	**Cancer**: J82, Scaber, 5637	Cancer lines: MEM Normal line: F-12 K	HS-SPME (DVB/PDMS) GC - SQ - IT - MS	2 ml sample + 0.59 g NaCl, pH = 2 & 7 5 min incubation 20 min extraction (45 °C, 250 rpm)	**Increased**: (1R,2 S,5R)-5-Methyl-2-(propan-2-yl)cyclo-hexan-1-ol, 1,3-Benzothiazole, 2,3-Dimethylhexane, 2-Methyl-2-pentanol, 2-Nonanone, 4-Methyl-3-penten-2-one, 4-Methyl-2-heptanone, 4-Methylnonane, 4-Methylpentan-2-one, 5-Octyloxolan-2-one, 6-Methyl-2-heptanone, Benzaldehyde, 1-Dodecanol, Dodecanal, Dodecane, Hexadecane, Naphthalene, Tetradecane, 2-Undecanone **Decreased**: (5Z)-6,10-Dimethylundeca-5,9-dien-2-one, Acetophenone, 1-Phenylethan-1-one, 2-(1R)-4-Methylcyclohex-3-en-1-ylpropan-2-ol, 2-Hydroxy-2-methylpropiophenone, 2-Methyl-2-heptanol, 2-Methyl-2-butanol, 2-Phenyl-2-propanol, 3-Methyl-1-butanol, 4-Methylbenzaldehyde, 1-Butanol, Cyclohexanone, Methyl benzoate, 1-Octanol, α-Methylstyrene
	**Normal**: SV-HUC-1				
Kidney: Amaro et al. (2020)	**Cancer**: 769-P, 786-O, ACHN, Caki-1, Caki-2	RPMI-1640	HS-SPME (DVB/CAR/PDMS) GC - SQ - IT - MS	2 ml sample + 0.43 g NaCl 11 min incubation 30 min extraction (44 °C, 250 rpm)	**Increased**: 2,4-Dimethyl-1-heptene, 2-Ethoxy-2-methylpropane, 2-Ethyl-1-hexanol, 3-Carene, 3-Methylbenzaldehyde, 4-Methylbenzaldehyde, Decane, Ethylbenzene, Formaldehyde, Styrene, Tetradecane, Xylene **Decreased**: 2,6-Dimethyl-7-octen-2-ol, Acetaldehyde, Acetone, Acetophenone, Benzaldehyde, Cyclohexanol, Cyclohexanone, Decanal, Dodecane, Levomenthol
	**Normal**: HK-2				

This table detailed the study, cell line used, culture media, sampling method, extraction condition, instrument platform and highlighted biomarkers. Compounds are arranged in alphabetical order

HS (headspace); SPME (solid-phase microextraction); CAR (Carboxen); DVB (Divinylbenzene); PDMS (Polydimethylsiloxane); SQ (single quadrupole), IT (ion trap)

It should be noted that all three cellular studies were conducted by the same research group using identical analytical platform, although the extraction conditions varied slightly. This consistency may improve technical reproducibility by reducing instrument-related variability, but it also limits external validation across different laboratories, instruments and experimental environments. In addition, repeated use of the same platform may increase the risk that certain recurrent VOCs reflect instrument-specific background signals, carryover, or laboratory-specific factors rather than universal biological changes.

The variation in extraction conditions may partly reflect the application of urine-optimized methods to cell culture media, despite the fact that urine and culture media are fundamentally different matrices and may require distinct sampling and extraction strategies. These findings emphasize the need for systematic optimization of cellular VOC sampling methods in future studies. Improved standardization and independent validation across laboratories will be essential for generating more robust mechanistic insights into cellular VOC production.

### Cross-matrix analysis of urinary and cellular VOC biomarkers

Since only one cellular study was identified for each cancer type, we first examined whether any VOCs were shared across prostate, bladder and kidney cancer cell-line studies (Table 5). Compounds detected repeatedly under different experimental conditions within the same study were counted only once.

Results show that 12 compounds were identified in at least two of the three cancer cell-line studies. Among these, six showed a consistent direction of change across cancer types, whereas the remaining six showed opposite or inconsistent trends (Table 5). Notably, Cyclohexanone was decreased in all three cancer cell-line studies compared with the respective control cell lines, suggesting that it may represent a common cellular VOC associated with altered metabolism in prostate, bladder and kidney cancer cells.

**Table 5:** Overlapping volatile organic compounds identified in cellular studies across different urological cancer cell lines

Compound	Prostate	Bladder	Kidney
2-(3-Methyl-3-cyclohexen-1-yl)-2-propanol	↓	↓	
Acetophenone		↓	↓
Cyclohexanone	↓	↓	↓
Methyl benzoate	↓	↓	
Naphthalene	↑	↑	
Tetradecane		↑	↑
1,3-Benzothiazole	↓	↑	
4-Methyl-2-heptanone	↓	↑	
4-Methyl-3-penten-2-one	↓	↑	
4-Methylbenzaldehyde	↓	↓	↑
Dodecane		↑	↓
Benzaldehyde		↑	↓

Arrows indicate the direction of change in VOC levels in cancer cell lines compared with their corresponding normal control lines (“↑” = increase; “↓” = decrease). Compounds are grouped according to the direction of change across cell-line studies and arranged alphabetically within each group

A similar organ-related pattern was observed in the cellular VOC studies. Ten of the 12 shared cellular VOCs were found between cancer cell lines derived from anatomically related organs, including prostate-bladder and bladder-kidney comparisons, regardless of whether the compounds were increased or decreased in cancer cells (Table 5). This pattern resembles the urine-based findings, in which greater VOC overlap was observed between anatomically or functionally adjacent organs. No compound was shared between prostate and kidney cancer cell lines. These findings suggest that, to some extent, cell-line VOC profiles may reflect organ-related metabolic similarities, even under controlled in vitro conditions. However, we again emphasize that this interpretation should remain cautious because only three cellular studies were available, all from the same research group, and differences in cell culture conditions, media composition and extraction methods may also influence the observed VOC profiles.

To further investigate the potential alignment between mechanistic cell-line studies and urine-based findings, we compared all VOC biomarkers identified in both cell culture media and urine samples. All significantly reported biomarkers were included in this comparison, regardless of whether they were increased, decreased, or reported without a clear direction of change. This analysis identified 28 compounds that overlapped between the two matrices (Table 6).

**Table 6:** Comparison of urinary and cellular volatile organic compound biomarkers

Compound	Prostate	Bladder	Kidney
**Concordant urinary and cellular trends**			
2-Ethyl-1-hexanol	∇	∇	Δ▲
4-Methyl-2-heptanone	▼	Δ▲	Δ
4-Methyl-2-pentanone		Δ▲	
5-Methyl-2-heptanone	∇▼		
Benzaldehyde		Δ▲	Δ▼
Dodecanal		Δ▲	
Tetradecane		▲	Δ▲
**Unclear or discordant urinary and cellular trends**			
2-Hydroxy-2-methylpropiophenone	∇	▼	
2,6-Dimethyl-7-octen-2-ol	**?**		▼
3-Carene	∇		▲
3-Methylbenzaldehyde	∇		▲
5-Methyl-2-isopropylcyclohexanol	▼		∇
Acetaldehyde	**?**	**?**	▼
Acetone		**?**	▼
Acetophenone	**?**	▼	▼
Benzyl acetate	▼	Δ	
Benzyl alcohol	▲	Δ	
Cyclohexanone	▼	Δ▼	▼
Decanal	**?**	∇	Δ▼
Decane			**?**▲
Dodecane		**?**▲	**?**▼
Ethylbenzene	Δ	Δ	▲
Formaldehyde		∇	▲
Hexadecane		**?**▲	
Methyl benzoate	Δ▼	▼	
Naphthalene	▲	**?**▲	∇
p-Xylene	∇▲		
Styrene			∇▲

Arrowhead symbols indicate the direction of VOC changes in cancer samples compared with their corresponding normal controls. Urinary VOC trends are shown as hollow arrowheads, with “Δ” = increased, “∇” = decreased, and “**?**” = unknown direction. Cellular VOC trends are shown as filled arrowheads, with “▲” = increased and “▼” = decreased. Compounds are grouped according to the agreement between urinary and cellular trends: concordant trends, or unclear/discordant trends. Within each group, compounds are arranged alphabetically

Seven compounds—2-Ethyl-1-hexanol, 4-Methyl-2-heptanone, 4-Methyl-2-pentanone, 5-Methyl-2-heptanone, Benzaldehyde, Dodecanal and Tetradecane—showed the same direction of change in both cellular and urinary studies of prostate, bladder or kidney cancers (Table 6). This cross-matrix consistency suggests that these compounds may have stronger potential as candidate VOC biomarkers, as they were detected in different biological matrices but showed concordant cancer-associated trends. These findings also indicate that in vitro cell models may, to some extent, reflect aspects of cancer-associated VOC metabolism observed in clinical urine samples.

The remaining 21 compounds showed less consistent cross-matrix patterns. Some were identified in the same cancer type in both cellular and urinary studies, but the direction of change was not reported in the urine studies. Others were detected in different cancer types or showed opposite trends between cellular and urinary samples. Therefore, these compounds should be interpreted more cautiously as cross-matrix biomarkers. Their inconsistent or incomplete trend information makes them less reliable for directly linking clinical urinary VOC findings with mechanistic studies using cell models.

## Discussion

In this review, we systematically categorized and compared VOC biomarkers detected in urine and cell culture media across prostate, bladder and renal cancers. We first identified cancer-specific urinary VOC patterns, including predominantly decreased organic acids and hydrocarbons in prostate cancer and more frequent elevations of ketones and aldehydes in bladder and renal cancers. We then examined recurrent urinary VOCs within each cancer type, which may represent more robust biomarker candidates for future diagnostic validation. In addition, “common VOC biomarkers” shared across cancer types were identified, suggesting potential shared metabolic alterations and, in some cases, possible physiological links between anatomically related organs within the urinary tract. Finally, by comparing urinary and cellular VOC profiles, we identified cross-matrix candidate biomarkers that may support future targeted validation and mechanistic investigation.

### Methodological heterogeneity and standardization needs

In this review, we identified recurrent VOC biomarkers within individual cancer types, across different cancer types, and across urine and cell culture media. The purpose of this comparison was not to generate statistically pooled biomarkers, but rather to highlight recurring compounds that may guide future targeted validation and mechanistic studies. However, it should be noted that these findings should be interpreted as a descriptive synthesis rather than a quantitative meta-analysis. This is important because VOC biomarker discovery is strongly influenced by methodological heterogeneity, including differences in sampling strategy, sorbent material, sample manipulation, analytical platform, data processing and statistical analysis. In addition, biological heterogeneity among patient cohorts further limits direct comparison across studies (Becker, 2020; Goertzen et al., 2024).

Three major sources of methodological heterogeneity are particularly relevant. First, instrumental and extraction-related variables, including the GC–MS platform, sampling mode and sorbent/coating material, may influence which VOC classes are preferentially detected. Second, sample-processing conditions, including salt addition, dilution and pH adjustment, can alter VOC partitioning and extraction efficiency. Third, data-processing and statistical strategies, including feature filtering, multiple-comparison correction and model-validation methods, may affect which compounds are ultimately reported as candidate biomarkers.

Regarding instrumental and extraction-related variability, although most included studies used GC–MS-based untargeted analysis for urinary or cellular VOC profiling, their sampling methods varied substantially. In particular, studies differed in their use of headspace versus immersive extraction, and in the choice of sorbent material, such as PDMS, CAR/PDMS, DVB/PDMS or CAR/DVB/PDMS. The number of VOCs reported across studies cannot be directly used to compare extraction efficiency, because each study used different preprocessing, thresholding and reporting criteria. Nevertheless, optimization studies provide useful methodological insights. Headspace extraction preferentially captures compounds that partition into the gas phase and reduces direct matrix contamination, whereas immersive extraction may improve recovery of less volatile compounds but can increase matrix effects. In food-related VOC studies, immersive extraction has been reported to yield more detectable VOCs, particularly lactones and less hydrophilic compounds (Cheng et al., 2021). However, urinary optimization studies suggest that, for urine samples, headspace extraction may provide more reliable VOC recovery and better overall performance (Myridakis et al., 2023; Wen et al., 2023). In addition, Myridakis et al. (2023) reported that the PDMS/CAR/DVB coating detected more than twice as many VOCs as PDMS alone in pooled urine samples. These findings emphasize that sampling mode and sorbent material should be optimized for the specific biological matrix and target VOC classes.

Second, sample manipulation can strongly affect VOC detection. Salt addition, dilution and pH adjustment may change the partitioning of VOCs between the liquid and gas phases and therefore alter extraction efficiency. For urine, salt (NaCl) addition is often used to increase sample osmolality and promote a salting-out effect, reducing VOC solubility in the aqueous phase and improving transfer into the headspace (Endo et al., 2012). Although some studies suggest that salt addition has only a limited effect on the total number of detected VOCs, specific compound classes, such as furans and esters, may be significantly affected (Cheng et al., 2021). In urine, which is an aqueous and relatively low-analyte matrix, salt addition is commonly used to improve VOC extraction efficiency (Yang et al., 2023). Wen et al. (2023) also showed that dilution reduced the detection of several key urinary VOCs, indirectly supporting the importance of matrix concentration and liquid–gas phase equilibrium in urinary VOC extraction.

pH adjustment is another important factor. Many urinary VOCs, including alcohols, aldehydes, ketones and organic acids, show improved detection under acidic conditions (Wen et al., 2023; Yang et al., 2023). Similarly, in cellular VOC studies, acidic extraction conditions have often been selected partly because they may resemble the acidic tumor microenvironment, but also because low pH can improve the detection of several VOC classes, including organic acids and esters (Rodrigues et al., 2018). However, pH effects are compound-dependent, and a condition that improves recovery of one VOC class may reduce the detection of another. Therefore, pH optimization should be performed according to the matrix type, study aim and expected chemical classes.

Beyond analytical platforms and sampling conditions, data-processing and statistical strategies also varied substantially across the included studies (Supplementary Table 4). This is particularly important for untargeted GC–MS-based volatolomics, where many VOCs or spectral features may be tested simultaneously. Several studies explicitly reported adjustment for multiple comparisons, including FDR correction (Heers et al. 2024; Lett et al. 2022; Monteiro et al. 2017; Pinto et al. 2021a, b; Riccio et al. 2023) or Bonferroni correction (Einoch Amor et al., 2023; Lima et al., 2019). However, formal multiple-testing correction was not consistently reported across all studies.

Many studies instead used feature filtering, removal of sporadic compounds (Gao et al., 2019), signal-to-noise filtering (Carapito et al., 2026), liberal *p*-value thresholds (Gao et al., 2019; Guest et al., 2021), LASSO/logistic regression (Badmos et al., 2024; Gao et al., 2019), machine-learning algorithms (Mao et al., 2025), cross-validation or independent validation cohorts. These approaches can support feature selection and reduce model overfitting, but they do not necessarily replace compound-level multiple-comparison correction. Therefore, individual VOC biomarkers, especially those reported in studies without clear FDR or Bonferroni adjustment, should be interpreted cautiously and prioritized for validation in independent cohorts.

Overall, the compound- and method-level synthesis presented in this review provides an initial framework for identifying recurrent VOC biomarkers and guiding future targeted studies. However, stronger clinical and mechanistic conclusions will require more standardized sampling conditions, matrix-specific method optimization, transparent data-processing workflows, appropriate multiple-comparison correction, and independent validation across cohorts and laboratories.

### Recurrent and common VOCs in urological cancers

In this review, the term “common biomarker” refers to a VOC reported in more than one urological cancer type or identified across both urine and cell culture media, rather than a biomarker proven to arise from the same tissue origin or biological pathway. This distinction is particularly important for cellular VOC studies because prostate, bladder and renal cancers originate from different cell types, including prostatic glandular epithelium, urothelium and renal tubular epithelium. In addition, even cell lines derived from the same cancer type may represent different biological contexts. For example, the prostate cell lines used by Lima et al. (2018) included a normal epithelial cell line (PNT2) and cancer cell lines derived from different metastatic sites, including bone metastasis (PC3), lymph node metastasis (LNCaP) and brain metastasis (DU145) (van Bokhoven et al., 2001). Therefore, no single cell line can fully represent an entire cancer type, and VOCs shared across cancer types should not be interpreted as evidence of a universal urological cancer mechanism. Nevertheless, cellular VOC studies remain valuable because they provide experimentally controllable systems for investigating mechanisms that are difficult to examine directly in clinical samples. These compounds are therefore best regarded as recurrent cross-cancer VOC candidates that require targeted validation and mechanistic investigation.

Although this review highlights recurrent VOCs as useful candidates for future targeted investigation, whether they are recurrent within one cancer type, shared across cancer types, or identified across matrices, the metabolic pathways underlying their production remain poorly understood. For instance, one of the overlapping compounds identified in this review, 2-Pentanone, has also been reported to increase significantly in other cancers such as breast and colorectal cancers (Porto-Figueira et al. 2018a, b), but its biochemical origin and regulatory mechanisms remain unexplored.

In contrast, dimethyl disulfide (DMDS) was found to decrease consistently in not only prostate and bladder prostate cancers (Cauchi et al., 2016; Ligor et al., 2022; Tyagi et al., 2021), but was also reported to decrease in lymphoma, leukemia, colorectal (Silva et al., 2011), breast (Silva et al., 2012), and lung (Priscilla Porto-Figueira et al. 2018a, b) cancers. Conversely, it appears to increase in head and neck cancers (Opitz & Herbarth, 2018) and has been associated with melanoma (Kwak et al., 2013). As a “common” biomarker, DMDS has been investigated mechanistically and has been implicated in its antioxidative role. It acts as a substrate for sulfoxide reductase A, helping to catabolize oxidants and reduce oxidative stress (Sa et al., 2021). Increased levels of DMDS may enhance this antioxidant effect in damaged cells, reducing lipid peroxidation (Sa et al., 2021). Therefore, the observed decrease in DMDS in prostate and bladder cancers could indicate a loss of this protective function, potentially contributing to increased cellular damage and carcinogenesis.

Given the limited mechanistic understanding of individual VOCs, pathway-level investigations that encompass broader category of VOCs is necessary. Previous review in prostate cancer identified five most abundant types of biomarkers – alcohols, aldehydes, organic acids, ketones, and hydrocarbons (Wen et al., 2020) (Supplementary Fig. 3), consistent with the findings summarized in this review. Among these, aldehydes (e.g., Hexanal and Decanal) are closely linked to lipid peroxidation, a process driven by reactive oxygen species (ROS). Elevated ROS induces oxidative cleavage of ω−6 fatty acids, generating a diverse range of saturated and unsaturated aldehydes that can diffuse into biofluids (Sutaria et al., 2022). In addition, metabolic enzymes such as alcohol dehydrogenase (ADH) and aldehyde dehydrogenase (ALDH), which regulate the interconversion and detoxification of these compounds, are frequently dysregulated in cancers (Puschel et al., 2021), making urinary aldehyde levels an integrated reflection of both oxidative stress and metabolic clearance efficiency.

Notably, ketones, such as 2-Butanone, 4-Heptanone and Cyclohexanone show significant alterations across cancer types in this review. Ketone-related metabolism plays an important role in tumor adaptation: under hypoxic or glucose-depleted conditions, cancer cells may oxidize ketone bodies via OXCT1 and ACAT1 to supply acetyl-CoA for the tricarboxylic acid (TCA) cycle, thereby supporting energy production and proliferation (Puchalska & Crawford, 2017; Rohena-Rivera et al., 2024). Aberrant ketone metabolism has also been associated with androgen-deprivation resistance in prostate cancer (Labanca et al., 2021) and chemoresistance in bladder cancer (Rohena-Rivera et al., 2024), supporting its potential biological and clinical relevance.

However, endogenous ketone bodies and excreted VOC ketones may be generated, consumed, or cleared through different metabolic routes. Therefore, changes in urinary VOC ketones may not be directly linked to altered ketone-body utilization, but may instead indicate broader changes in cancer-associated energy metabolism, oxidative stress, or urinary excretion. Further targeted studies are needed to determine whether VOC ketones are mechanistically linked to ketone-body metabolism or represent related but distinct metabolic outputs.

Collectively, these findings suggest that altered VOC profiles in urological cancers may reflect distinct but partially overlapping metabolic processes, including redox imbalance, lipid peroxidation, altered aldehyde/alcohol metabolism and ketone-associated energy adaptation. By identifying recurrent VOCs within individual cancer types, shared VOCs across cancer types, and overlapping VOCs across urine and cell culture media, this review highlights candidate compounds with greater potential for reproducibility and biological relevance. The inclusion of cellular VOC data may provide a useful bridge between clinical biofluid observations and future mechanistic studies, although these cross-matrix candidates still require targeted validation in independent cohorts and controlled experimental models before they can be considered clinically reliable biomarkers.

### Current diagnostic limitations and potential advantages of VOC biomarkers

Despite advances in imaging and biomarker testing, current diagnostic approaches for urological cancers continue to face significant limitations. Prostate cancer is a clear example of how traditional methods often fall short of delivering accurate, patient-friendly early detection.

Invasive procedures remain central to diagnosis. Techniques such as DRE, serum PSA testing, TRUS and biopsy not only cause discomfort but can also lead to complications including infections, urinary dysfunction, and even long-term impacts such as erectile dysfunction (Thompson et al., 2007). While non-invasive imaging methods like MRI scans offer improved anatomical detail, they rarely stand alone; multiple diagnostic modalities are typically required to increase accuracy, which further increases cost and complexity (Cornford et al., 2024).

Even with this multi-modal approach, accuracy remains a major concern. Tests such as DRE and PSA lack the sensitivity and specificity required to reliably detect early-stage disease. For instance, elevated PSA levels are not exclusive to prostate cancer and can be observed in benign conditions such as prostatic hyperplasia, resulting in high false-positive rates (Madu & Lu, 2010). This, in turn, exacerbates a larger problem: overdiagnosis. Patients undergo unnecessary biopsies and years of follow-up monitoring, often without any benefit to survival outcomes, but with significant impact on quality of life (Madu & Lu, 2010).

Cost and time are equally challenging. MRI scans and other imaging procedures are expensive and time-consuming, often requiring anesthesia or multiple clinic visits. Even after sampling, tissue biopsies and serum marker analyses involve lengthy processing times and subjective interpretation by medical professionals, which introduces variability and potential bias (Cornford et al., 2024).

Taken together, these limitations highlight the continued need for more reliable, efficient, and patient-friendly diagnostic approaches. VOC biomarkers offer a promising complementary strategy because they can be measured non-invasively, cost-effectively, and may capture biologically informative metabolic changes (Wen et al., 2020). With appropriate standardization and validation, VOC analysis could help reduce unnecessary invasive procedures, lower diagnostic burden and improve early detection, thereby benefiting both patients and healthcare systems.

### Diagnostic performance of VOC-based models

Current prostate cancer diagnostic pathways usually begin with risk assessment based on PSA levels, DRE, family history and other clinical factors, followed by MRI and biopsy where indicated (Cornford et al., 2024). However, PSA testing remains limited by imperfect sensitivity and specificity, and biopsy remains invasive. Therefore, non-invasive approaches that can improve diagnostic discrimination remain clinically important.

As summarized in Table 1, several VOC-based diagnostic models have achieved sensitivity and specificity exceeding 90% in different urological cancers, suggesting that VOC profiling could provide a non-invasive alternative to current approaches. Using prostate cancer as an example, PSA testing is less invasive than biopsy but suffers from limited specificity and sensitivity. Several studies have directly compared PSA-based and VOC-based diagnostic models. For instance, Khalid et al. (2015) demonstrated that their random forest model achieved higher accuracy (74%) than PSA testing (63–65%). Similarly, Gao et al. (2019) validated their VOC-based model in an independent patient cohort, achieving an impressive 92% diagnostic accuracy, representing a 38% improvement over traditional PSA testing (54%) in the same cohort. Collectively, these findings demonstrate the strong potential of VOC-based diagnostics to enhance early detection and reduce unnecessary invasive procedures.

Over the past decade, the reported diagnostic performance of VOC-based models appears to have improved. However, direct comparison across studies should be made cautiously because of differences in cohort design, analytical platform, validation strategy and model construction. This apparent improvement may partly reflect advances in analytical instrumentation, improved VOC detection sensitivity, more standardized data-processing workflows and the increasing use of machine-learning-based model construction. For example, two studies enrolled similar number of patients (approximately 100) demonstrated marked improvements on diagnostic accuracy – from 74% in 2015 (Khalid et al., 2015) to 96% in 2023 (Liu et al., 2023). This trend reflects the rapid technological progress and methodological standardization achieved in recent VOC research, indicating a promising trajectory toward clinical translation.

Beyond diagnostic accuracy, analytical robustness is also important for VOC biomarker development. GC–MS remains widely used in VOC analysis because it provides sensitive detection, chromatographic separation and compound-level identification across biological matrices, including urine. For example, Guest et al. (2021) demonstrated that while trained canines achieved diagnostic accuracies of 71% sensitivity and 76% specificity in distinguishing cancer from control urine samples, the refined GC-MS–based VOC model built on the same cohort achieved 85.4% sensitivity and 100% specificity, which highlights the superior analytical precision and reproducibility of GC-MS platforms compared with biological olfactory approaches in biomarker discovery.

### Current limitations in VOC studies

Despite considerable progress, VOC biomarker research still faces several persistent limitations. The included studies differed substantially in clinical cohort design, sample collection, storage, sample processing, extraction strategy, analytical platform, data preprocessing and statistical analysis. Therefore, the recurrent and shared VOCs identified in this review should be interpreted as a descriptive synthesis rather than statistically pooled biomarkers. Because of this heterogeneity, and because raw datasets were not consistently available, quantitative meta-analysis or post hoc statistical adjustment could not be performed.

One major source of heterogeneity is pre-analytical sample handling and VOC extraction. Sample manipulation, including salt addition, dilution and pH adjustment, can alter VOC partitioning between the liquid and gas phases and affect extraction efficiency. Similarly, extraction mode and sorbent material, such as headspace versus immersive extraction and PDMS versus PDMS/CAR/DVB coatings, may preferentially recover different VOC classes. These factors make it difficult to determine whether differences in reported biomarkers reflect true biological variation or methodological effects.

A second limitation concerns quality control, compound identification, data processing and statistical analysis. Although many studies used blanks, QC samples, internal standards and NIST library matching, these procedures were not uniformly applied or reported. Compound identification based mainly on spectral library matching, without confirmation using retention indices, authentic standards or other orthogonal methods, may reduce confidence in individual VOC assignments. In addition, only some studies reported FDR or Bonferroni correction, whereas others relied on feature filtering, liberal *p*-value thresholds, multivariate modelling, machine-learning algorithms, cross-validation or independent validation cohorts. These approaches can support feature selection and reduce overfitting, but they do not fully replace formal compound-level multiple-comparison correction. Environmental contamination, instrument carryover and exogenous VOC sources were also not always systematically assessed, which is particularly important for low-abundance urinary VOC analysis.

Biological variability is another important challenge in urinary VOC studies. Diet, medication, smoking status, renal function, microbiota composition, inflammation and other comorbidities may all influence urinary VOC profiles. These factors were not consistently controlled or reported across the included studies, making it difficult to distinguish cancer-associated VOCs from host-, environment- or lifestyle-related signals.

Finally, cellular VOC studies remain at an early stage, and cross-matrix comparisons between cell culture media and urine should be interpreted cautiously. Only one cellular VOC study was identified for each cancer type, limiting the robustness of cross-cancer comparisons. Cell-line models also cannot fully represent the biological complexity of prostate, bladder or renal cancers, which arise from different tissues and cellular origins. Culture conditions, especially medium composition, serum use, oxygen tension and pH, can strongly influence VOC production. For example, Rodrigues et al. (2018) compared bladder cancer and normal cell lines cultured in different media, introducing matrix-specific bias that could distort VOC profiles. Therefore, procedures for cell culture medium collection, storage and VOC extraction should be systematically optimized and tested to establish more comparable and broadly applicable workflows for cellular VOC studies, which would support future mechanistic investigations. In addition, urine and cell culture media are fundamentally different matrices: urinary VOCs may reflect not only tumor metabolism, but also renal filtration, urinary storage, microbiota, inflammation, systemic metabolism and environmental exposure. Therefore, VOCs overlapping between cell culture media and urine should be considered exploratory candidates for mechanistic validation rather than confirmed biomarkers.

A limitation of the review process should also be acknowledged. This review was not prospectively registered; however, detailed search strategies, eligibility criteria, PRISMA flowcharts, quality assessment and data-extraction tables are provided to improve transparency and reproducibility.

Addressing these limitations will require harmonized protocols, matrix-specific optimization, larger multi-center cohorts, independent validation, transparent reporting of preprocessing and statistical workflows, and stronger integration of clinical biofluid studies with controlled mechanistic experiments.

## Conclusions

Volatile organic compound profiling represents an emerging and increasingly important field in analytical oncology. Through this systematic review, we integrated evidence from urinary and cellular studies across prostate, bladder and renal cancers, identifying distinct yet overlapping VOC signatures that may reflect both cancer-specific metabolic reprogramming and shared urological physiology. The identification of recurrent and cross-matrix biomarker candidates, including aldehydes, ketones and organic acids, highlights the potential biochemical relevance of oxidative stress, lipid peroxidation and altered energy metabolism in these malignancies. Although methodological heterogeneity and limited cohort sizes still constrain reproducibility, continued advances in analytical precision, data processing and model validation are improving the reliability of VOC-based biomarker discovery. These developments, combined with the growing integration of mass spectrometry, machine learning and mechanistic cell-based models, position VOC analysis as a promising bridge between metabolomics and clinical diagnostics.

Looking forward, harmonizing sampling protocols, establishing large-scale multi-center studies, and coupling VOC readouts with genetic, proteomic or other molecular markers will be crucial for clinical translation. Nonetheless, the collective progress to date supports the feasibility of VOC biomarkers as a non-invasive, cost-effective and physiologically informative tool for the early detection and monitoring of urological cancers. With continued methodological refinement and rigorous validation, VOC analysis may become a valuable component of future precision oncology.

## Supplementary Information

Below is the link to the electronic supplementary material.

Supplementary material 1 (DOCX 998.9 kb)

## Data Availability

All data extracted and analyzed in this review are available in the main manuscript tables and Supplementary Information. No new primary experimental datasets or analytic code were generated in this study.
