# Supplementary material for: Urinary and cellular volatile organic compounds as biomarkers for urological cancers: a systematic review of GC-MS-based volatolomics
Source: Metabolomics. 2026 Jul 27;22(4):133. doi: 10.1007/s11306-026-02493-7 (PMC13407726; doi:10.1007/s11306-026-02493-7)
Supplement: Supplementary file 1 — Supplementary material 1 (DOCX 998.9 kb) [file 11306_2026_2493_MOESM1_ESM.docx]

**Supplementary Information for**

**Urinary and cellular volatile organic compounds as biomarkers for urological cancers: a systematic review of GC-MS-based volatolomics**

Boyu Xie^1,3^, Qing Wen^2,3^

^1^Faculty of Biophysics, Biochemistry and Molecular Biology, School of Life Sciences, Tsinghua University, Beijing 100084, China

^2^Department of Urology, The First Affiliated Hospital, School of Medicine, Zhejiang University, Hangzhou 310000, China

^3^Department of Surgery and Cancer, Imperial College London, London W12 0HS, UK

To whom correspondence should be addressed:

Email: [boyu.xie@imperial.ac.uk](mailto:boyu.xie@imperial.ac.uk)

Supplementary Table 1. PubMed search strategies used for urinary and cellular VOC studies in urological cancers, together with supplementary broad search terms for urological or genitourinary cancers. Search terms were constructed using controlled vocabulary, including Medical Subject Headings (MeSH), and free-text terms related to cancer type, VOCs, and sample matrix or experimental model. Boolean operators were used to combine terms, with “OR” applied within each concept block and “AND” applied between concept blocks. Equivalent search concepts were adapted for Embase, Web of Science and Scopus according to each database interface, while Google Scholar was used as a supplementary source for exploratory checking.

| Urinary VOC Search Terms | | |
| --- | --- | --- |
| Prostate cancer studies | **Bladder cancer studies** | **Renal cancer studies** |
| (  "Prostatic Neoplasms"[Mesh]  OR "prostate cancer"[Title/Abstract]    OR "prostatic cancer"[Title/Abstract]    OR "prostatic neoplasm*"[Title/Abstract]    OR "prostatic carcinoma"[Title/Abstract]    OR "prostatic adenocarcinoma"[Title/Abstract]  )  AND  (  "Volatile Organic Compounds"[Mesh]    OR "volatile organic compound*"[Title/Abstract]    OR VOC[Title/Abstract]    OR VOCs[Title/Abstract]    OR volatilome[Title/Abstract]    OR volatolome[Title/Abstract]    OR volatolomics[Title/Abstract]    OR volatilomics[Title/Abstract]    OR "volatile metabolite*"[Title/Abstract]  )  AND  (  urine[Title/Abstract]    OR urinary[Title/Abstract]     OR headspace[Title/Abstract]  ) | (  "Bladder Neoplasms"[Mesh]  OR "bladder cancer"[Title/Abstract]  OR "bladder carcinoma"[Title/Abstract]  OR "bladder neoplasm*"[Title/Abstract]  OR "urothelial carcinoma"[Title/Abstract]  OR "transitional cell carcinoma"[Title/Abstract]  )  AND  (  "Volatile Organic Compounds"[Mesh]  OR "volatile organic compound*"[Title/Abstract]  OR VOC[Title/Abstract]  OR VOCs[Title/Abstract]  OR volatilome[Title/Abstract]  OR volatolome[Title/Abstract]  OR volatolomics[Title/Abstract]  OR volatilomics[Title/Abstract]  OR "volatile metabolite*"[Title/Abstract]  )  AND  (  urine[Title/Abstract]  OR urinary[Title/Abstract]  OR headspace[Title/Abstract]  ) | (  "Kidney Neoplasms"[Mesh]  OR "kidney cancer"[Title/Abstract]  OR "renal cancer"[Title/Abstract]  OR "renal neoplasm*"[Title/Abstract]  OR "renal carcinoma"[Title/Abstract]  OR "renal cell carcinoma"[Title/Abstract]  )  AND  (  "Volatile Organic Compounds"[Mesh]  OR "volatile organic compound*"[Title/Abstract]  OR VOC[Title/Abstract]  OR VOCs[Title/Abstract]  OR volatilome[Title/Abstract]  OR volatolome[Title/Abstract]  OR volatolomics[Title/Abstract]  OR volatilomics[Title/Abstract]  OR "volatile metabolite*"[Title/Abstract]  )  AND  (  urine[Title/Abstract]  OR urinary[Title/Abstract]  OR headspace[Title/Abstract]  ) |
| Cellular VOC Search Terms | | |
| Prostate cancer studies | **Bladder cancer studies** | **Renal cancer studies** |
| (  "Prostatic Neoplasms"[Mesh]  OR "prostate cancer"[Title/Abstract]  OR "prostatic cancer"[Title/Abstract]  OR "prostatic neoplasm*"[Title/Abstract]  OR "prostatic carcinoma"[Title/Abstract]  OR "prostatic adenocarcinoma"[Title/Abstract]  )  AND  (  "Volatile Organic Compounds"[Mesh]  OR "volatile organic compound*"[Title/Abstract]  OR VOC[Title/Abstract]  OR VOCs[Title/Abstract]  OR volatilome[Title/Abstract]  OR volatolome[Title/Abstract]  OR volatolomics[Title/Abstract]  OR volatilomics[Title/Abstract]  OR "volatile metabolite*"[Title/Abstract]  )  AND  (  "cell line*"[Title/Abstract]  OR cellular[Title/Abstract]  OR "cell culture"[Title/Abstract]  OR exometabolome[Title/Abstract]  OR headspace[Title/Abstract]  ) | (  "Bladder Neoplasms"[Mesh]  OR "bladder cancer"[Title/Abstract]  OR "bladder carcinoma"[Title/Abstract]  OR "bladder neoplasm*"[Title/Abstract]  OR "urothelial carcinoma"[Title/Abstract]  OR "transitional cell carcinoma"[Title/Abstract]  )  AND  (  "Volatile Organic Compounds"[Mesh]  OR "volatile organic compound*"[Title/Abstract]  OR VOC[Title/Abstract]  OR VOCs[Title/Abstract]  OR volatilome[Title/Abstract]  OR volatolome[Title/Abstract]  OR volatolomics[Title/Abstract]  OR volatilomics[Title/Abstract]  OR "volatile metabolite*"[Title/Abstract]  )  AND  (  "cell line*"[Title/Abstract]  OR cellular[Title/Abstract]  OR "cell culture"[Title/Abstract]  OR exometabolome[Title/Abstract]  OR headspace[Title/Abstract]  ) | (  "Kidney Neoplasms"[Mesh]  OR "kidney cancer"[Title/Abstract]  OR "renal cancer"[Title/Abstract]  OR "renal neoplasm*"[Title/Abstract]  OR "renal carcinoma"[Title/Abstract]  OR "renal cell carcinoma"[Title/Abstract]  )  AND  (  "Volatile Organic Compounds"[Mesh]  OR "volatile organic compound*"[Title/Abstract]  OR VOC[Title/Abstract]  OR VOCs[Title/Abstract]  OR volatilome[Title/Abstract]  OR volatolome[Title/Abstract]  OR volatolomics[Title/Abstract]  OR volatilomics[Title/Abstract]  OR "volatile metabolite*"[Title/Abstract]  )  AND  (  "cell line*"[Title/Abstract]  OR cellular[Title/Abstract]  OR "cell culture"[Title/Abstract]  OR exometabolome[Title/Abstract]  OR headspace[Title/Abstract]  ) |

**Supplementary Broad Urological/Genitourinary Cancer Search Terms**

| (  "urological cancer*"[Title/Abstract]  OR "urologic cancer*"[Title/Abstract]  OR "urological neoplasm*"[Title/Abstract]  OR "urologic neoplasm*"[Title/Abstract]  OR "urological malignanc*"[Title/Abstract]  OR "urologic malignanc*"[Title/Abstract]  OR "genitourinary cancer*"[Title/Abstract]  OR "genitourinary neoplasm*"[Title/Abstract]  OR "genitourinary malignanc*"[Title/Abstract]  AND  (  "Volatile Organic Compounds"[Mesh]  OR "volatile organic compound*"[Title/Abstract]  OR VOC[Title/Abstract]  OR VOCs[Title/Abstract]  OR volatilome[Title/Abstract]  OR volatolome[Title/Abstract]  OR volatolomics[Title/Abstract]  OR volatilomics[Title/Abstract]  OR "volatile metabolite*"[Title/Abstract]  OR "VOC biomarker*"[Title/Abstract]  OR "volatile biomarker*"[Title/Abstract]  )  AND  (  urine[Title/Abstract]  OR urinary[Title/Abstract]  OR "cell line*"[Title/Abstract]  OR cellular[Title/Abstract]  OR "cell culture"[Title/Abstract]  OR exometabolome[Title/Abstract]  OR headspace[Title/Abstract]  ) |
| --- |

Supplementary Table 2. PRISMA 2020 checklist for this systematic review. The table lists each PRISMA item, the corresponding checklist requirement, and the location in the manuscript or Supplementary Information where the item is reported. Items not applicable to this review, such as quantitative meta-analysis, sensitivity analysis, formal reporting-bias assessment, or protocol amendments, are indicated as “Not applicable” or “Not formally assessed” where appropriate.

| **Section and Topic** | **Item #** | **Checklist item** | **Location where item is reported** |
| --- | --- | --- | --- |
| **TITLE** | | |  |
| Title | 1 | Identify the report as a systematic review. | Title page, Abstract, Introduction **1.4**, Methods **2.1** |
| **ABSTRACT** | | |  |
| Abstract | 2 | See the PRISMA 2020 for Abstracts checklist. | The abstract contains Background, Aim of Review, Key Scientific Concepts of Review |
| **INTRODUCTION** | | |  |
| Rationale | 3 | Describe the rationale for the review in the context of existing knowledge. | Introduction section **1.1-1.3** |
| Objectives | 4 | Provide an explicit statement of the objective(s) or question(s) the review addresses. | Introduction section **1.4**, final paragraph |
| **METHODS** | | |  |
| Eligibility criteria | 5 | Specify the inclusion and exclusion criteria for the review and how studies were grouped for the syntheses. | Methods section **2.2**, paragraphs describing urinary and cellular VOC inclusion/exclusion criteria |
| Information sources | 6 | Specify all databases, registers, websites, organisations, reference lists and other sources searched or consulted to identify studies. Specify the date when each source was last searched or consulted. | Methods section **2.1**, first paragraph; Supplementary Table 1 |
| Search strategy | 7 | Present the full search strategies for all databases, registers and websites, including any filters and limits used. | Methods section **2.1**, second and third paragraphs; Supplementary Table 1 |
| Selection process | 8 | Specify the methods used to decide whether a study met the inclusion criteria of the review, including how many reviewers screened each record and each report retrieved, whether they worked independently, and if applicable, details of automation tools used in the process. | Methods section **2.2**, Zotero/deduplication paragraph and reviewer-screening paragraph; Figure 1; Supplementary Figures 1–2 |
| Data collection process | 9 | Specify the methods used to collect data from reports, including how many reviewers collected data from each report, whether they worked independently, any processes for obtaining or confirming data from study investigators, and if applicable, details of automation tools used in the process. | Methods section **2.4**, first paragraph; Supplementary Table 4 |
| Data items | 10a | List and define all outcomes for which data were sought. Specify whether all results that were compatible with each outcome domain in each study were sought (e.g. for all measures, time points, analyses), and if not, the methods used to decide which results to collect. | Methods section **2.4**, first and fourth paragraphs; Tables 1–6 |
|  | 10b | List and define all other variables for which data were sought (e.g. participant and intervention characteristics, funding sources). Describe any assumptions made about any missing or unclear information. | Methods section **2.4**, first paragraph; Supplementary Table 4 |
| Study risk of bias assessment | 11 | Specify the methods used to assess risk of bias in the included studies, including details of the tool(s) used, how many reviewers assessed each study and whether they worked independently, and if applicable, details of automation tools used in the process. | Methods section **2.3**; Supplementary Table 3 |
| Effect measures | 12 | Specify for each outcome the effect measure(s) (e.g. risk ratio, mean difference) used in the synthesis or presentation of results. | Methods section **2.4**, final paragraph; Table 1; Tables 2–6 |
| Synthesis methods | 13a | Describe the processes used to decide which studies were eligible for each synthesis (e.g. tabulating the study intervention characteristics and comparing against the planned groups for each synthesis (item #5)). | Methods section **2.4**, paragraphs on VOC biomarker selection and descriptive synthesis; Results sections **3.1–3.5** |
|  | 13b | Describe any methods required to prepare the data for presentation or synthesis, such as handling of missing summary statistics, or data conversions. | Methods sections **2.4** and **2.5**; Supplementary Table 4 |
|  | 13c | Describe any methods used to tabulate or visually display results of individual studies and syntheses. | Methods section **2.4**; Tables 1–6; Figures 1–2; Supplementary Figures 1–4 |
|  | 13d | Describe any methods used to synthesize results and provide a rationale for the choice(s). If meta-analysis was performed, describe the model(s), method(s) to identify the presence and extent of statistical heterogeneity, and software package(s) used. | Methods section **2.4**, descriptive synthesis/no meta-analysis paragraph |
|  | 13e | Describe any methods used to explore possible causes of heterogeneity among study results (e.g. subgroup analysis, meta-regression). | Discussion sections **4.1** and **4.5** |
|  | 13f | Describe any sensitivity analyses conducted to assess robustness of the synthesized results. | Not applicable; no sensitivity analysis was conducted |
| Reporting bias assessment | 14 | Describe any methods used to assess risk of bias due to missing results in a synthesis (arising from reporting biases). | Only discussed as limitations in Discussion sections **4.1** and **4.5**; Supplementary Table 4 records statistical/reporting details |
| Certainty assessment | 15 | Describe any methods used to assess certainty (or confidence) in the body of evidence for an outcome. | Only methodological quality/risk of bias assessed using QUADAS-2 in Methods section **2.3** and Supplementary Table 3 |
| **RESULTS** | | |  |
| Study selection | 16a | Describe the results of the search and selection process, from the number of records identified in the search to the number of studies included in the review, ideally using a flow diagram. | Results section **3.1**; Figure 1; Supplementary Figures 1–2 |
|  | 16b | Cite studies that might appear to meet the inclusion criteria, but which were excluded, and explain why they were excluded. | Figure 1; Supplementary Figures 1–2 |
| Study characteristics | 17 | Cite each included study and present its characteristics. | Table 1 for urinary studies; Table 4 for cellular studies; Results sections **3.1** and **3.4** |
| Risk of bias in studies | 18 | Present assessments of risk of bias for each included study. | Supplementary Table 3 |
| Results of individual studies | 19 | For all outcomes, present, for each study: (a) summary statistics for each group (where appropriate) and (b) an effect estimate and its precision (e.g. confidence/credible interval), ideally using structured tables or plots. | Table 1 for urinary studies; Table 4 for cellular studies |
| Results of syntheses | 20a | For each synthesis, briefly summarise the characteristics and risk of bias among contributing studies. | Results sections **3.1-3.5**; Tables 1–6; Supplementary Table 3 |
|  | 20b | Present results of all statistical syntheses conducted. If meta-analysis was done, present for each the summary estimate and its precision (e.g. confidence/credible interval) and measures of statistical heterogeneity. If comparing groups, describe the direction of the effect. | Not applicable; no quantitative meta-analysis was performed. Descriptive synthesis is presented in Results sections **3.1–3.5** and Tables 1–6 |
|  | 20c | Present results of all investigations of possible causes of heterogeneity among study results. | Discussion sections **4.1** and **4.5** |
|  | 20d | Present results of all sensitivity analyses conducted to assess the robustness of the synthesized results. | Not applicable; no sensitivity analysis was conducted |
| Reporting biases | 21 | Present assessments of risk of bias due to missing results (arising from reporting biases) for each synthesis assessed. | Only discussed in Discussion sections **4.1** and **4.5** |
| Certainty of evidence | 22 | Present assessments of certainty (or confidence) in the body of evidence for each outcome assessed. | Risk of bias/ methodological quality summarized in Supplementary Table 3 and discussed in Discussion section **4.5** |
| **DISCUSSION** | | |  |
| Discussion | 23a | Provide a general interpretation of the results in the context of other evidence. | Discussion opening paragraph; Discussion sections **4.1–4.4** |
|  | 23b | Discuss any limitations of the evidence included in the review. | Discussion section **4.5** |
|  | 23c | Discuss any limitations of the review processes used. | Discussion section **4.5**, paragraph noting that the review was not prospectively registered |
|  | 23d | Discuss implications of the results for practice, policy, and future research. | Discussion sections **4.3–4.5**; Conclusions |
| **OTHER INFORMATION** | | |  |
| Registration and protocol | 24a | Provide registration information for the review, including register name and registration number, or state that the review was not registered. | Discussion section **4.5**: review was not prospectively registered |
|  | 24b | Indicate where the review protocol can be accessed, or state that a protocol was not prepared. | Discussion section **4.5**: no prospectively registered protocol; transparency supported by detailed methods and supplementary materials |
|  | 24c | Describe and explain any amendments to information provided at registration or in the protocol. | Not applicable; no registered protocol was prepared |
| Support | 25 | Describe sources of financial or non-financial support for the review, and the role of the funders or sponsors in the review. | Acknowledgments |
| Competing interests | 26 | Declare any competing interests of review authors. | Competing Interests section |
| Availability of data, code and other materials | 27 | Report which of the following are publicly available and where they can be found: template data collection forms; data extracted from included studies; data used for all analyses; analytic code; any other materials used in the review. | Data availability statement, Supplementary Tables 1–4; Tables 1–6; Reference list |

(A)


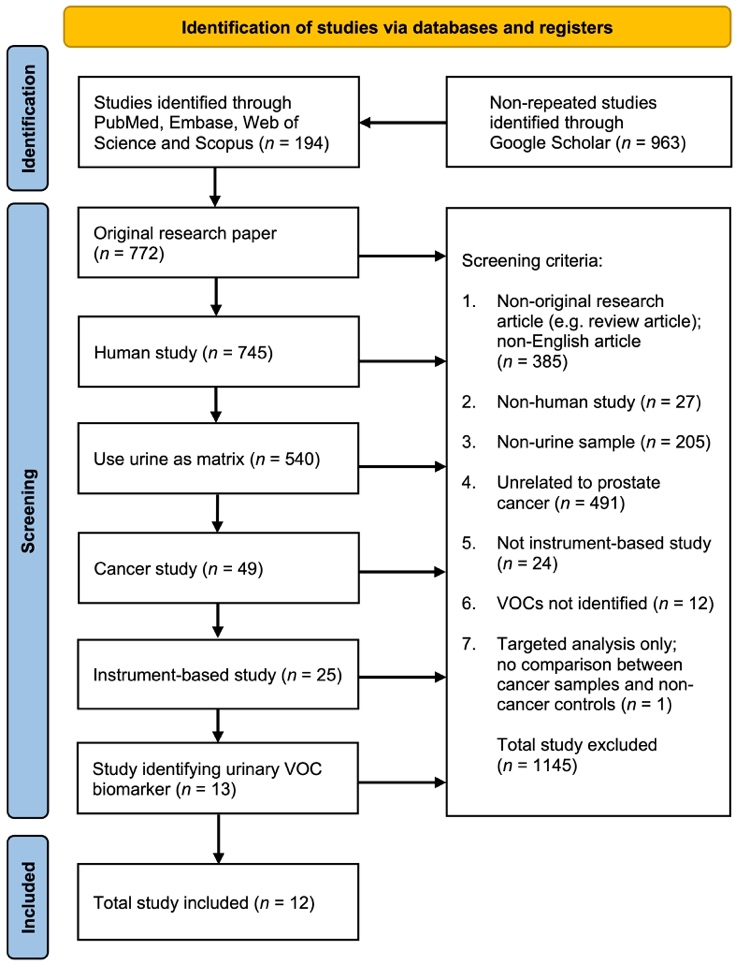


(B)


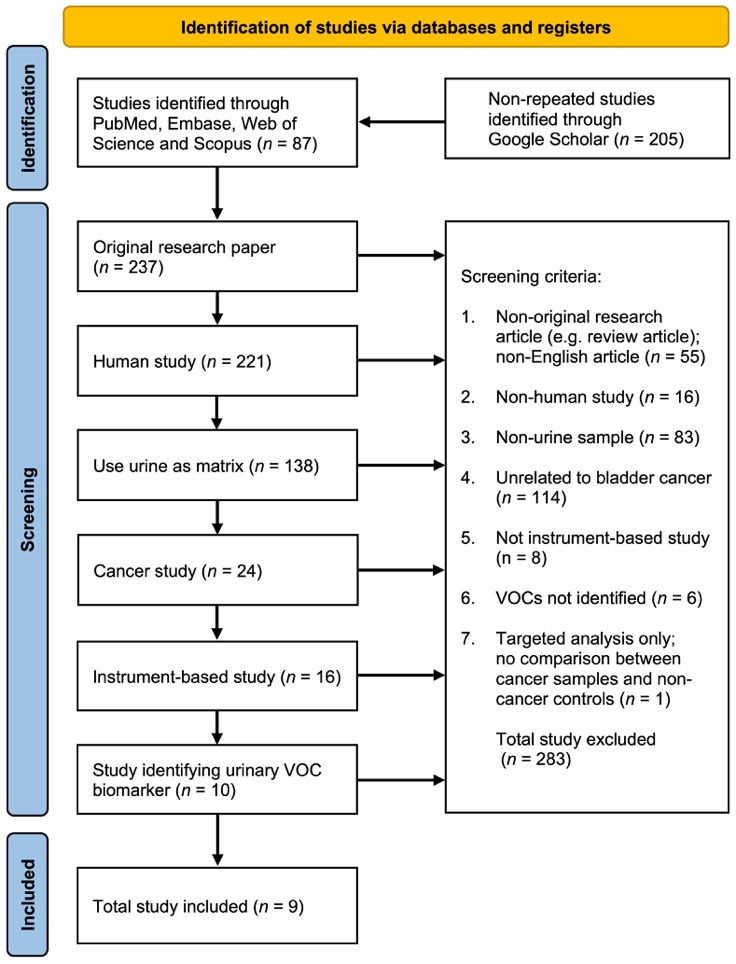


(C)


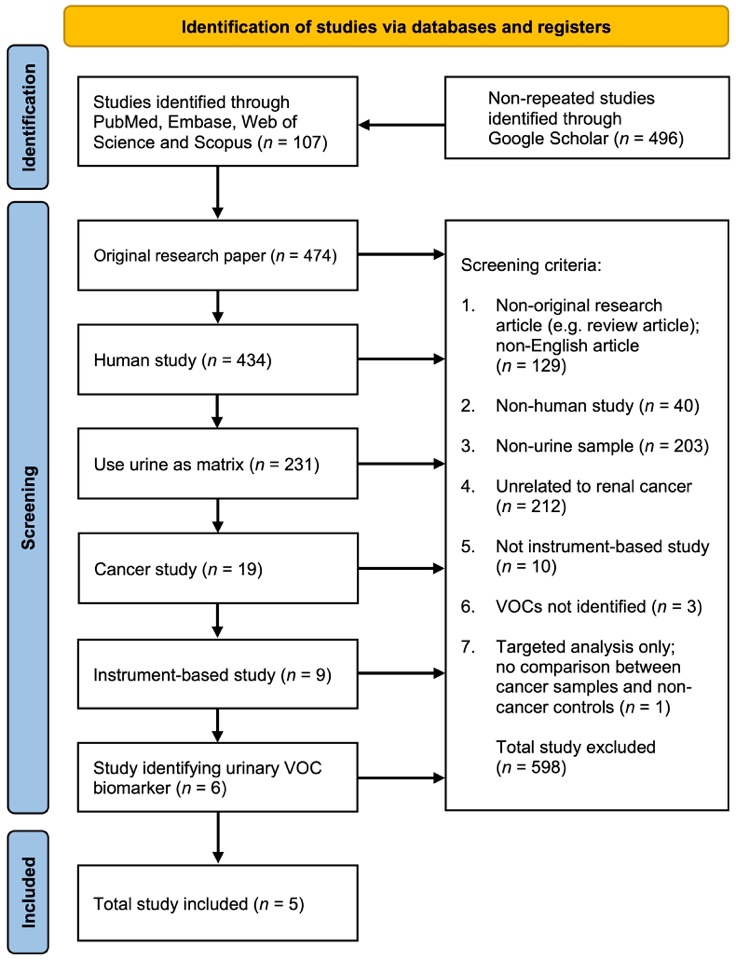


Supplementary Figure 1. Preferred Reporting Items for Systematic Reviews and Meta-Analyses (PRISMA) flowchart for the systematic review of urinary VOC studies in (A) prostate, (B) bladder, and (C) renal cancers. It details the systematic process of data search, study identification, and screening criteria applied for inclusion in this review.


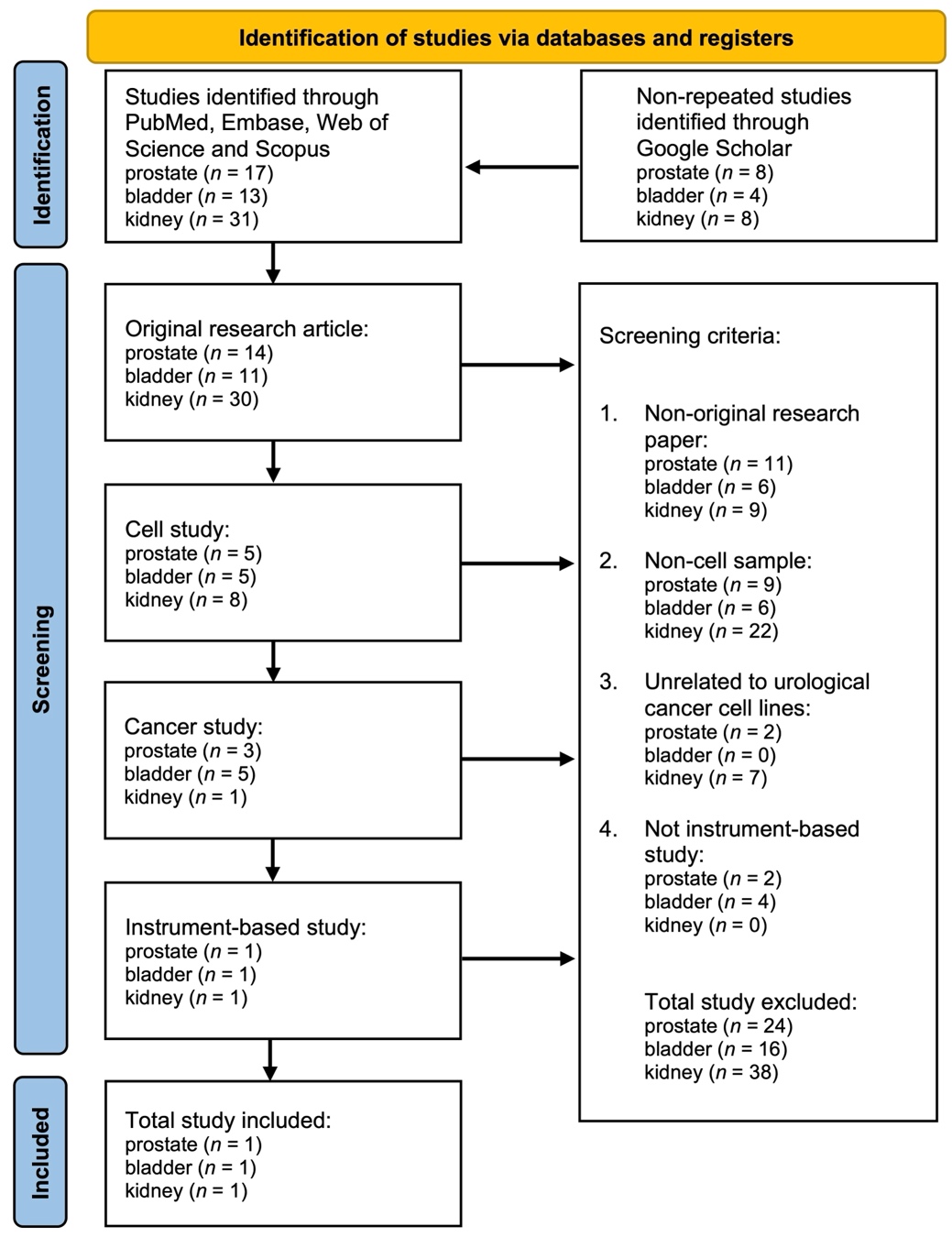


Supplementary Figure 2. Preferred Reporting Items for Systematic Reviews and Meta-Analyses (PRISMA) flowchart for the systematic review of cellular VOC studies in prostate, bladder and kidney cancers. The diagram illustrates the systematic process of database searching, study identification, and screening criteria applied for inclusion in this review.

Supplementary Table 3. Methodological quality assessment (risk of bias and applicability) of studies using the Quality Assessment of Diagnostic Accuracy Studies (QUADAS-2) tool across four domains: patient selection, index test, reference standard, and flow and timing. The signaling questions were tailored in advance for VOC diagnostic studies, and the assessment form was piloted before use. Two reviewers also independently evaluated each study, with disagreements resolved by consensus. Domain-level judgements were recorded as “Low”, “High”, or “Not Reported”, following QUADAS-2 guidance.

| Study | Risk of Bias | | | | Applicability Concerns | | |
| --- | --- | --- | --- | --- | --- | --- | --- |
| Prostate cancer studies | | | | | | | |
|  | **Patient selection** | **Index test** | **Reference standard** | **Flow and timing** | **Patient selection** | **Index test** | **Reference standard** |
| Smith et al. (2010) | High | Low | Low | Low | High | Low | Low |
| Khalid et al. (2015) | Low | Low | High | Low | Low | Low | Low |
| Struck-Lewicka et al. (2015) | Low | Low | Not Reported | Low | Low | High | Low |
| Jiménez-Pacheco et al. (2018) | High | Low | Low | Low | Low | High | Low |
| Gao et al. (2019) | Low | Low | Low | Low | Low | Low | Low |
| Lima et al. (2019) | High | Low | Low | Low | Low | Low | Low |
| Lima et al. (2020) | High | Low | Low | Low | Low | Low | Low |
| Guest et al. (2021) | High | Low | Low | Low | Low | Low | Low |
| Tyagi et al. (2021) | Low | Low | Low | Low | Low | Low | Low |
| Liu et al. (2023) | Low | Low | Low | Low | Low | Low | Low |
| Riccio et al. (2023) | Low | Low | Low | Low | Low | Low | Low |
| Badmos et al. (2024) | Low | Low | Low | Low | Low | Low | Low |
| Bladder cancer studies | | | | | | | |
| Jobu et al. (2012) | High | Low | Low | Low | High | Low | Low |
| Cauchi et al. (2016) | Low | Low | Low | Low | Low | Low | Low |
| Pinto, Carapito, et al. (2021) | Low | Low | Low | Low | Low | Low | Low |
| Tyagi et al. (2021) | Low | Low | Low | Low | Low | Low | Low |
| Lett et al. (2022) | Low | Low | Low | Low | Low | Low | Low |
| Ligor et al. (2022) | Low | Low | Low | Low | Low | Low | Low |
| Heers et al. (2024) | Low | Low | Low | Low | Low | Low | Low |
| Mao et al. (2025) | Low | Low | Low | Low | Low | Low | Low |
| Carapito et al. (2026) | Low | Low | Low | Low | Low | Low | Low |
| Renal cancer studies | | | | | | | |
| Wang et al. (2016) | Low | Low | Low | High | Low | Low | Low |
| Monteiro et al. (2017) | Low | Low | Low | Low | Low | Low | Low |
| Pinto, Amaro, et al. (2021) | Low | Low | Low | Low | Low | Low | Low |
| Einoch Amor et al. (2023) | Low | Low | Low | Low | Low | Low | Low |
| Holbrook et al. (2024) | High | Low | Low | High | Low | Low | Low |

Supplementary Table 4. Summary of analytical, data-processing and statistical strategies employed across the included studies. The parameters evaluated include: 1) use of **internal standards**, recorded as “Yes” or “Not reported”; 2) **compound** **identification methods**, including retention time (RT) or retention index (RI), or equivalent approaches; 3) **library matching**, including the National Institute of Standards and Technology (NIST) library or comparable spectral databases; 4) **use of blanks or quality-control (QC) samples** and other data-screening methods, such as match factor (MF) or reverse match factor (RMF); 5) **data processing and feature-screening strategies**, including dataset trimming prior to analysis, dynamic baseline correction (DBC), chromatographic alignment, exclusion of low-confidence or unqualified compounds, signal-to-noise ratio (S/N)-based filtering, multiple-comparison correction where reported, such as false discovery rate (FDR) correction, Bonferroni correction, liberal *p*-value thresholding, and Least Absolute Shrinkage and Selection Operator (LASSO) regression; 6) **statistical analysis**, including multivariate and univariate approaches, with specific univariate tests shown where reported.

| Studies | Internal Standard | Identification Methods (RI/RT) | Library Matching | QC/blank samples | Data Processing Strategies | Statistical Analysis |
| --- | --- | --- | --- | --- | --- | --- |
| Prostate cancer studies | | | | | | |
| Smith et al. (2010) | Not reported | RT | NIST 05 | Not reported | Simple matching coefficient, distributions of the similarity coefficient | t-tests |
| Khalid et al. (2015) | Not reported | RT | NIST 11 | MF> 800 & RMF> 800 | Deconvolution, MF> 800, rule out of sporadic compounds | Multivariate analysis |
| Struck-Lewicka et al. (2015) | Yes | RT | NIST 11 | QC/blank | Coefficient of variation>50% | Multivariate analysis + t-test + Mann-Whitney U test |
| Jiménez-Pacheco et al. (2018) | Not reported | RT | NIST (version not reported) | Not reported | Pearson correlation | Multivariate analysis + ANOVA |
| Gao et al. (2019) | Yes | RT | NIST (version not reported) | Not reported | Removal of sporadic features detected in <3% of samples, liberal *p*-value cutoff of 0.2, logistic regression and LASSO regression modelling | Multivariate analysis + Wilcoxon rank-sum test |
| Lima et al. (2019) | Yes | RT | NIST 14 | QC/blank, 6.5-38 min, m/z range 50-600 | Baseline normalization, deconvolution and alignment, total peak area normalization, Bonferroni correction | Multivariate analysis + Mann-Whitney U test |
| Lima et al. (2020) | Yes | RT | NIST 14 | QC/blank, including only 6.5-38 min, m/z range 50-600 | Baseline normalization, deconvolution and alignment | Multivariate analysis + Mann-Whitney U test + Student’s t-test |
| Guest et al. (2021) | Not reported | RT | NIST 17 | QC/blank, RMF>600 | Deconvolution, removal of sporadic features (<3% of samples), liberal *p*-value cutoff of 0.2 | Multivariate analysis + Wilcoxon rank-sum test |
| Tyagi et al. (2021) | Not reported | RT | NIST 11 | Not reported | Peak area thresholding, removal of background noise and sporadic VOCs | Multivariate analysis + Wilcoxon rank-sum test |
| Liu et al. (2023) | Not reported | RI | NIST (version not reported) | QC/blank | Peak height > 3 times baseline noise | Mann-Whitney U test |
| Riccio et al. (2023) | Yes | RT | NIST 05 | QC/blank, Similarity threshold >80% | Cube-root transformation, mean-centered scaling, FDR correction | Multivariate analysis + t-test |
| Badmos et al. (2024) | Yes | RT | NIST 17 | RMF>500 | Two-sample t-test, simple logistic regression, LASSO regression | Multivariate analysis + Wilcoxon rank-sum test |
| Bladder cancer studies | | | | | | |
| Jobu et al. (2012 | Not reported | RT | NIST (version not reported) | QC/blank | DBC, compiling, deconvolution | Multivariate analysis |
| Cauchi et al. (2016 | Yes | RT | NIST and MassBank | QC/blank | Removal of signals m/z > 33 | Multivariate analysis |
| Pinto, Carapito, et al. (2021) | Yes | RI | NIST 14 | QC/blank, RMF>770 | Baseline normalization, deconvolution and alignment, FDR correction | Multivariate analysis + Mann-Whitney U test |
| Tyagi et al. (2021) | Not reported | RT | NIST 11 | Not reported | Peak area thresholding, removal of background noise and sporadic VOCs | Multivariate analysis + Wilcoxon rank-sum test |
| Lett et al. (2022) | Not reported | RT | NIST 11 | QC/blank | Deconvolution, data alignment, RT/fragment-ion matching check, FDR correction (adjusted *p* <0.05) | Multivariate analysis + Wilcoxon rank-sum test |
| Ligor et al. (2022) | Not reported | RT | NIST 11 | MF>850 | Baseline normalization, peak thresholding, signal-to-noise ratio thresholding, deconvolution | Kolmogorov–Smirnov test |
| Heers et al. (2024) | Not reported | Ion migration time | NIST (version not reported) | Not reported | Friedman’s test for continuous and Fisher’s exact test for categorical variables, FDR correction | Mann-Whitney U test, chi-square test, t-test |
| Mao et al. (2025) | Not reported | Ion migration time | Not reported | Not reported | Machine-learning algorithms (details not reported) | Multivariate analysis + Mann-Whitney U test |
| Carapito et al. (2026) | Yes | RT + RI | NIST 14 | QC/blank | Data alignment, total peak area normalization, S/N-based filtering (<10), logistic regression | Multivariate analysis + Wilcoxon rank-sum test |
| Renal cancer studies | | | | | | |
| Wang et al. (2016) | Not reported | RT | NIST 11 | Not reported | Total peak area normalization | Multivariate analysis + t-test |
| Monteiro et al. (2017) | Not reported | RT + RI | NIST 14 | QC/blank, MF>800 & RMF>800 | Data alignment, FDR correction (adjusted *p* < 0.05) | Multivariate analysis |
| Pinto, Amaro, et al. (2021) | Yes | RT | NIST 14 | QC/blank | Baseline normalization, deconvolution and alignment, peak thresholding, FDR correction (adjusted *p* < 0.05) | Multivariate analysis + Mann-Whitney U test |
| Einoch Amor et al. (2023) | Yes | RT | NIST 14 | Not reported | Bonferroni correction, hierarchical clustering, machine-learning algorithms | Multivariate analysis + Wilcoxon rank-sum test |
| Holbrook et al. (2024) | Yes | RT | NIST 17 | QC/blank | Removal of sporadic features (in <3% of samples), removal of compounds outside the dataset interquartile range (>40%), liberal *p*-value cutoff of 0.2 | Multivariate analysis + Wilcoxon rank-sum test |


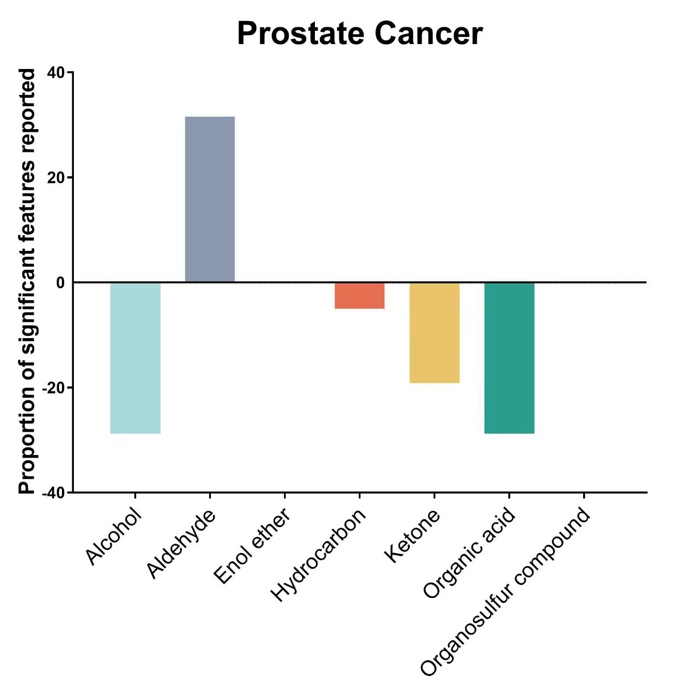


Supplementary Figure 3. Distribution of compound classes identified in prostate cancer VOC studies from a previous review (adapted from Wen et al., 2020).


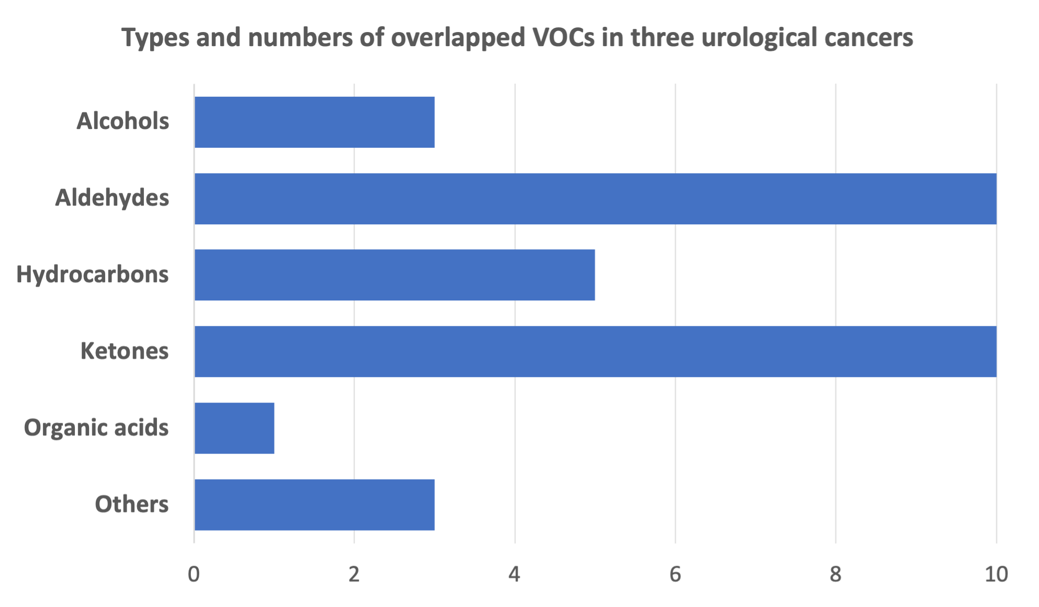


Supplementary Figure 4. Distribution of compound classes among overlapping volatile organic compounds identified in urine samples from patients with prostate, bladder or renal cancers. Bars represent the number of overlapping compounds. Compound classes include alcohols, aldehydes, hydrocarbons, ketones, organic acids, and others.
